# Defining and Dividing the Greater Caribbean: Insights from the Biogeography of Shorefishes

**DOI:** 10.1371/journal.pone.0102918

**Published:** 2014-07-23

**Authors:** D. Ross Robertson, Katie L. Cramer

**Affiliations:** Smithsonian Tropical Research Institute, Balboa, Panamá; California Polytechnic State University, United States of America

## Abstract

The Greater Caribbean biogeographic region is the high-diversity heart of the Tropical West Atlantic, one of four global centers of tropical marine biodiversity. The traditional view of the Greater Caribbean is that it is limited to the Caribbean, West Indies, southwest Gulf of Mexico and tip of Florida, and that, due to its faunal homogeneity, lacks major provincial subdivisions. In this scenario the northern 2/3 of the Gulf of Mexico and southeastern USA represent a separate temperate, “Carolinian” biogeographic region. We completed a comprehensive re-assessment of the biogeography of the Greater Caribbean by comparing the distributions of 1,559 shorefish species within 45 sections of shelf waters of the Greater Caribbean and adjacent areas. This analysis shows that that the Greater Caribbean occupies a much larger area than usually thought, extending south to at least Guyana, and north to encompass the entire Carolinian area. Rather than being homogenous, the Greater Caribbean is divided into three major provinces, each with a distinctive, primarily tropical fauna: (1) a central, tropical province comprising the West Indies, Bermuda and Central America; (2) a southern, upwelling-affected province spanning the entire continental shelf of northern South America; and (iii) a northern, subtropical province that includes all of the Gulf of Mexico, Florida and southeastern USA. This three-province pattern holds for both reef- and soft bottom fishes, indicating a general response by demersal fishes to major variation in provincial shelf environments. Such environmental differences include latitudinal variation in sea temperature, availability of major habitats (coral reefs, soft bottom shorelines, and mangroves), and nutrient additions from upwelling areas and large rivers. The three-province arrangement of the Greater Caribbean broadly resembles and has a similar environmental basis to the provincial arrangement of its sister biogeographic region, the Tropical Eastern Pacific.

## Introduction

The delimitation of the world’s faunal regions has been a major focus of marine biogeography since its beginnings in the mid-nineteenth century (e.g., [1]). These and later analyses [2]–[5] identified four major centers of tropical marine biodiversity: the Indo-west Pacific, the tropical eastern Pacific (TEP), the tropical west Atlantic (TWA) and the tropical east Atlantic. The TWA includes two large, geographically well separated areas of shelf that contain an abundance of coral and rocky reef habitat, the Greater Caribbean (the Caribbean and immediately adjacent areas to the north and south) and Brazil [5], [6]. The reef faunas of those two areas are partly isolated by a broad expanse of soft bottom shoreline produced by enormous freshwater outflows between the Orinoco and Amazon rivers. While these two regions share many species of reef fishes and other shorefishes, each also has a substantial number of regional endemic shorefishes [7], [8]. Compared to Brazil, the Greater Caribbean (GC) has almost twice the number of species and twice the rate of regional endemism among reef fishes [6], and thus represents the high-diversity heart of the TWA. Assessments of the geographic limits and subdivisions of the GC have long been part of marine biogeographic studies, and have included analyses not only of whole faunas [3], [9], [10] but also of specific taxa (mainly invertebrates [2]; bivalves and gastropods [4]; mainly fishes [5], [11]; decapod crustaceans [12]; and a selection of macro-gastropods [13]).

**Figure 1:**
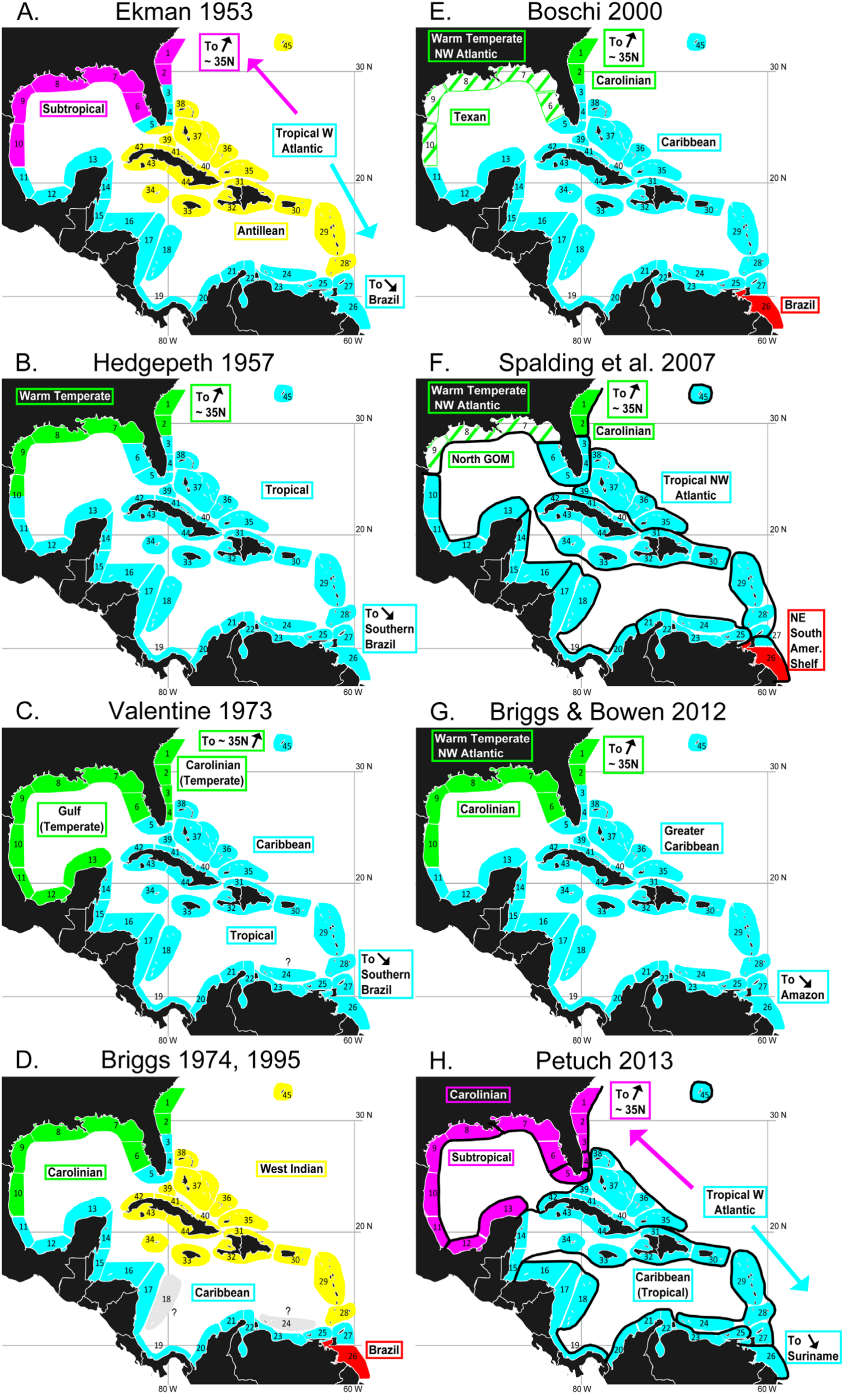
Previous schemes for the biogeographic structure of the study area. Schemes produced by previous authors (2–5, 9–12). These are based on maps in each publication except for (2), whose scheme was reconstructed from the text.

**Figure 2:**
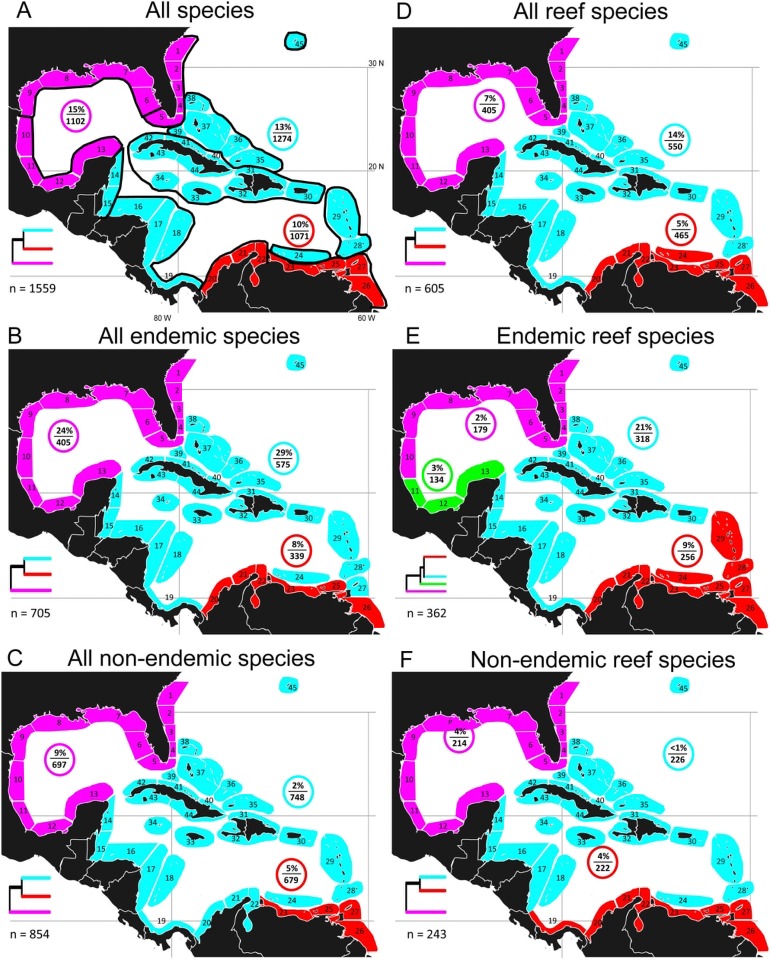
Optimal configuration of major clusters of sites: whole fauna and reef fishes. Optimal configuration of major clusters of sites based on betasim dissimilarity dendrograms and evaluation curve fitting A: whole fauna, colored areas indicate 3 major clusters; %/n in colored circle indicates % of species unique to that cluster and no. species in the cluster; n = total number of species; each dendrogram is a schematic based on the corresponding whole dendrogram in Figure S3, indicating relationships between the major clusters; black lines outline sub-clusters based on cutting the dendrogram in Fig. S3A at 1/3 the maximum beta-sim value (see text). B–F cluster patterns for subsets of the whole fauna: endemic and non-endemic members of the whole fauna, reef fishes (all, endemic, non-endemic).

**Figure 3:**
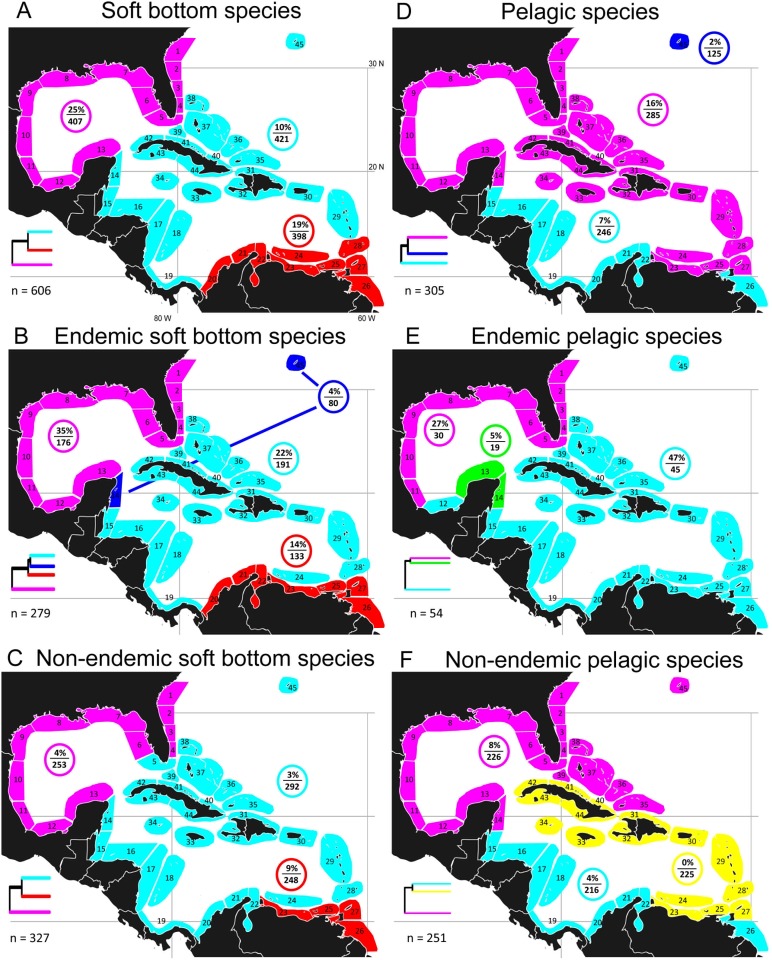
Optimal configuration of major clusters of sites: soft bottom species and pelagic species. Optimal major clusters of sites based on beta-sim dissimilarity dendrograms and evaluation curve fitting (see methods). A–F cluster patterns for subsets of the whole fauna. A–C: all soft bottom species, and their endemic and non-endemic members; D–F: all pelagic species, and their endemic and non-endemic members; %/n in colored circle indicates % of species unique to that cluster and no. species in the cluster; n = total number of species; each dendrogram is a schematic based on the corresponding whole dendrogram in Figure S4, indicating relationships between the major clusters.

Eight biogeographic schemes for the GC produced over the past 60 years (Fig. 1) are in large part derived by reviews and synthesis of the scientific literature. None of them involved quantitative analyses of the detailed distributions of many species throughout a large area that included not only the GC but also adjacent areas to its north and south. These schemes generally divided the Caribbean and surrounding areas into two major biogeographic units: (1) a “Caribbean” unit that comprises the entire Caribbean plus the West Indies, Bermuda and the southern tip of Florida and is characterized by tropical sea surface temperatures (SSTs) as well as a clearly tropical biota that includes an abundance of reef building corals, and (2) a “Gulf” unit centered on the Gulf of Mexico that has cooler winter SSTs and includes a significant number of temperate species that also occur northwards along the eastern US coast. The geographical limits of the Gulf unit and its zoogeographic relationship to the Caribbean unit vary among the different schemes. Some authors [3]–[5], [10], [11] considered the Gulf unit to be a temperate biogeographic unit distinct from and equivalent in rank to the tropical Caribbean unit, and included only the southwest Gulf of Mexico and/or lower parts of the Florida peninsula as part of the Caribbean unit (Fig. 1B–D, F, G). Others [2], [13], however, regarded the Gulf unit as a subtropical part of the TWA, along with the tropical Caribbean unit (Fig. 1A, H). Secondary levels of biogeographic subdivision proposed for this general area include (1) possible separation of West Indian and mainland Caribbean areas ([2], [5], [9], but see [11]; Fig. 1A, D, G), (2) separation of the northern Gulf of Mexico and the Atlantic coast of the US into two sub-units of a single temperate unit [10], [12]; see Fig. 1E, F) or two separate temperate units ([4]; see Fig. 1C), and (3) division of the present study area into about 12 small faunal sub-units ([10], [13]; Fig. 1E, H). The boundaries inside the Gulf of Mexico between the Gulf unit and the Caribbean units also vary among these different schemes, as do the southern limits of the Caribbean unit and the nature of its faunal relationship to the Brazilian unit of the TWA (Fig. 1A–H).

Methods for defining biogeographic units and their subdivisions vary. Some studies focus exclusively on patterns of endemism [5], [9], [11]–[13]. However, others consider the general distinctiveness of biotas, a method with a long history of use (c.f. [14]) that has been employed in both regional [15], [16] and global analyses [10], [17], [18]. Faunal distinctiveness incorporates information on geographic patterns of occurrence of all members of regional and local faunas, including by not limited to endemics, which frequently represent a minor component of a fauna. Distinctiveness can be used to establish a hierarchy of relationships among local faunas that define boundaries between biogeographic units (c.f. [15]) without relying on arbitrary “rules” based on endemism levels [11] or external factors such as SST [11], [18], and avoiding the influence of preconceptions arising from previous biogeographic assessments. This general-similarity approach also allows for an explicit assessment of the influence of endemic species by examination of spatial patterns of both regional endemics and non-endemics [19].

In this study, we present the first quantitative assessment of the geographic limits and sub-divisions of the Greater Caribbean based on an analysis of primary-source data on the detailed distributions of species belonging to a large, diverse and ecologically important taxon: its shallow-water shorefish fauna. We take a similar approach to defining the geographic limits and faunal subdivisions of the GC that we used for the TEP [19], analyzing the taxonomic and ecological distinctiveness of local shorefish faunas in different parts of the Caribbean and adjacent areas as well as the geography of local endemism. We then briefly compare the biogeographic structure of the GC to that of its sister biogeographic region, the TEP, an adjacent tropical area that shared a long common history with the GC before becoming separated by the rise of the Central American Isthmus.

For the purposes of this study, “regions” are major geographic centers of endemism and distinctiveness (e.g., the Greater Caribbean), “provinces” are major subdivisions of a region with their own distinctive subsets of the regional fauna, and a species may be both a regional and provincial endemic if it is restricted to a particular province within a region. To simplify the discussion, we do not include the northwest coast of Cuba as part of the Gulf of Mexico, which we treat as being entirely continental.

Determining the limits and subdivisions of biogeographic regions and analysis of similarities of local faunas within regions can be useful in two ways. First, such information helps to identify the contemporary and historical environmental forces that produce patterns of similarity among faunas at different spatial scales. Second, it can inform management strategies at both regional and local scales by identifying the extent of faunal connectivity between parts of regions and pinpointing areas with different faunal assemblages. This study focuses on the former issue, although our results have some implications for the latter.

## Methods

### The study area and its geographic partitioning

The study area includes the continental and insular shelves of the West Atlantic between central South Carolina (33° N) and northern Guyana (7° N). This area is geographically highly complex, with a long winding continental shoreline, an abundance of large and small islands, and large scattered areas of island-free insular shelf. Many major environmental discontinuities occur over very short distances in this area, e.g., the mainland and islands on each side of the narrow straits of Florida. Subdividing the study area using a 1-degree latitude/longitude grid (cf [19], [20]) would result in grid-cells spanning such discontinuities at various locations. In addition, the shorefish faunas of many of the several hundred sites that would be produced by such a procedure are poorly known (see Fig. S1). Instead, we subdivided the study area into 45 sections of the continental and insular shelves, with boundaries between those sections based on environmental discontinuities likely strong enough to affect the composition of local fish assemblages. Such discontinuities included the mouths of large rivers, coastal upwelling zones, edges of zones with different temperature or climate regimes, major changes in benthic habitat types, and continental and insular locations well separated by deep water.

The island of Aruba off western Venezuela was not included in our analyses. While the rest of the southwest Antilles off northern South America are oceanic islands well separated by deep water from the continental shelf, Aruba is on the continental shelf, near the mouth of the large, brackish Gulf of Venezuela. Hence, that island likely experiences environmental conditions different to those affecting the remainder of the southwest Antilles. In addition, the shorefish fauna of Aruba is very poorly known.

### The components of the fish fauna included in the analyses

We included 1,559 species of elasmobranchs and bony fishes that are apparent residents in the study area and which live in the upper 100 m of the water column of continental and insular shelves. Residency was indicated by their restriction to the study area, an abundance of site records in our database, or comments in the literature about their regional population status. Species that live exclusively below 100 m were not included because their geographic distributions are much less well known and are likely affected by different environmental factors to those influencing shallow-water species.

In addition to analyzing the distributions of the entire fauna we also assessed those of members of five sub-groups. These included three groups based on habitat usage: reef fishes (those found only on hard bottoms or that rely on shelter of hard bottoms), soft bottom species (those restricted to sand and mud bottoms in marine or estuarine habitats), and pelagic species (restricted entirely or largely to the water column). Species were assigned to different habitat groups with no overlap in group membership. Due to inadequate habitat information a small number of species (<50) in our database were not included in the above three groups. The two other subgroups assessed were exclusively marine species and non-marine species restricted to brackish and freshwater habitats. Within each of these groups, we also performed separate analyses on Greater Caribbean regional endemics and non-endemics. Regional endemics included species that have at least 75% of their geographic ranges within the area bounded by central eastern Florida and Bermuda in the north and Trinidad in the south, with no more than minor range extensions along the continental shelf immediately beyond those boundaries. All other species were classed as regional non-endemics (e.g., circumtropical species, or TWA endemics that also occur in Brazil).

### Geographic distribution database

We compiled distributional data from both primary and secondary sources (see Appendix S1). Primary sources included databases of geo-referenced collection records from global aggregators and museums, universities, and research organizations that have become publically available on the internet over the last decade. In addition, we extracted information from many scientific publications containing original descriptions of species, reviews of genera, and species lists for particular locations or areas. The latter included the Gulf of Mexico, the Atlantic coast of Mexico, Belize, Cuba, Bermuda, Puerto Rico, the Bahamas, St Croix, Colombia, Curacao and Venezuela. We also incorporated information from general regional or subregional treatments of the fish fauna (see Appendix S1). Additional unpublished distributional data on specific taxa or sites was provided by individual researchers. Data were also obtained from collecting expeditions aimed at documenting shorefish distributions that were conducted by DRR and collaborators between 2004–2010 along both coasts of Florida, as well as Mississippi, Louisiana, Texas, the Bahamas, Panama, Curacao, and sites scattered along the coast and continental islands of Venezuela between the mouth of the Orinoco River and the mouth of the Gulf of Venezuela.

Additional information on species distributions was obtained from a series of workshops in which DRR participated that were organized by the International Union for the Conservation of Nature (IUCN) to review the IUCN Red List status of the members of the Greater Caribbean shorefish fauna. Taxonomic experts at these meetings reviewed information on the geographic ranges of almost all of the species considered here, allowing us to construct and refine the species range maps in our database. The resulting species maps were sufficiently fine grained to incorporate disjunctions in distributions such as those known for various temperate species found in the northern Gulf of Mexico and northern Florida, but not southern Florida (e.g., [8]). Some range maps were truncated to take into account the fact that the Gulf Stream carries juveniles of some tropical species well beyond the northern limits of adult ranges, where they die off during winter [21].

Museum databases continue to be plagued by often substantial geo-referencing errors [22] that have yet to be resolved either by their primary sources or by global aggregators. Hence, records from museum databases were mapped, and isolated records of common species located far outside their known ranges or in inappropriate habitat (e.g, littoral demersal species at deep ocean locations) were deleted. This process, which included the removal of likely duplicate records, reduced the database by 18%, leaving ∼800,000 species site records (see Fig. S1).

### Analytical methods

Biogeographic patterns within the GC were determined using a hierarchical cluster analysis of dissimilarities in species composition (presence/absence) among the 45 sites. Clustering was performed on a dissimilarity matrix that was produced by computing the beta-sim dissimilarity index for each pairing of the 45 sites. We chose beta-sim because it is appropriate for presence-absence data, does not treat joint absences as evidence of similarity between groups, and, unlike the popular Bray-Curtis metric, is not sensitive to differences in species richness [23], [24]. Kulbicki et al. [18] also used beta-sim in their recent hierarchical cluster analysis of global reef fish distributions.

The optimal number of major biogeographic clusters was determined using a procedure [17] that assessed variation in the average proportion of “local species” (species occurring in only one cluster) that resulted from cutting each cluster dendrogram at a range of possible numbers of clusters. Such local species include both GC endemics and non-endemics. We refer to this combined group as “local species” to distinguish them from GC endemics. The proportion of local species was selected as the evaluation metric because it is directly related to the degree of species turnover or beta diversity across biogeographic units. An evaluation curve was created by plotting the average proportion of local species per cluster against all likely numbers of clusters (see below). The optimal number of clusters was determined from the location of the “knee” of the evaluation curve – the point of maximum curvature, at which an increase in the number of clusters is no longer associated with a steep decline in the proportion of local species (e.g., Fig. S2). The knee of the evaluation curve was determined by the “L-method” algorithm developed by Salvador and Chan [25], which identifies the knee of an L-shaped curve by finding the intersection point between the two straight lines that most closely fit the curve. The location of the knee was computed by taking into account the root mean squared error for the best-fit line to the left and right of each possible number of clusters. The optimal number of clusters was determined by minimizing the total root mean squared error (RMSEc) from the equation:
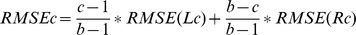
Where *b* = maximum number of clusters considered, *c* = number of clusters where the possible knee of the curve is located, *RMSE(L_c_)* = root mean squared error for the straight line to the left of *c,* and *RMSE(R_c_)* = root mean squared error for the straight line to the right of *c*. To ensure an L-shaped curve and to a avoid evaluating unrealistically fine cluster partitions [25], the maximum number of clusters considered was determined by cutting off the clustering dendrogram at 75% of its maximum beta-sim value. Because the L-method requires that each fitted line contains at least three points, the minimum number of clusters evaluated by this method was three. This method of defining larger scale partitions of the study area avoids arbitrary rules about cut-off levels of similarity or endemism, and facilitates comparisons of subdivision patterns of different subsets of a fauna, regardless of quantitative differences in dissimilarity levels among different analyses. This empirical method allows the data to define the pattern, ensuring objectivity in defining biogeographic boundaries.

Although we used hierarchical cluster analyses of faunal similarity in both this study and our study of the biogeography of the shorefishes of the TEP [19], we used different methods to determine the optimal number and composition of major clusters. For the TEP study, clustering was based on the Bray-Curtis dissimilarity metric, and the major-cluster membership of different sites was then refined using an analysis of variance permutation technique (ANOSIM). For the present study clustering was based on the beta-sim dissimilarity metric and the number of major clusters was determined using the local species evaluation curve. Two factors could lead to different outcomes from these different approaches: (1) beta-sim values are not affected by differences in fauna size, while Bray-Curtis values are, and (2) ANOSIM assesses faunal similarity only while the evaluation curve method assesses unique species occurrences (of local species) within clusters. To assess the possible differences in regional limits and major-cluster configurations resulting from the two different approaches, we analyzed the GC whole-fauna dataset using three combinations of methods: (1) Bray-Curtis/ANOSIM, (2) beta-sim/ANOSIM, and (3) beta-sim/evaluation curve.

## Results

For the entire fauna, the beta-sim/evaluation curve analysis indicated an optimum number of three clusters for the study area (Figs. 2A, S3A). Regional endemics, representing 45.2% of the fauna, demonstrated the same tripartite subdivision (Fig. 2B, S3B). These three clusters included: (1) a central section comprising the central American coast from Mexico to Panama, plus all the offshore islands (the West Indies (except the continental islands of Trinidad and Tobago) and Bermuda); (2) a northern, continental section consisting of all the Gulf of Mexico plus the Atlantic coast of the US north to South Carolina, and (3) the entire continental shelf of northern South America from Colombia to northern Guyana. The regional non-endemics (54.8% of the fauna) showed a similar pattern, but with the western half of northern South America as part of the central section. In all three cases the most divergent fauna was that of the northern section (see dendrogram summaries in Figs. 2A–C, full dendrograms in Figs. S3A–C). This section had the highest proportion of “local” species (i.e., those unique to a cluster) in two of those three cases (see Figs. 2A–C, S3A–C). All three clusters had higher proportions of “local” species within the regional endemic assemblage than the non-endemic assemblage (Figs. 2B, C, Table S1). In the all species, all endemics, and all non-endemics analyses, at least 85% of the species present in one cluster also occurred in one or both of the other two clusters (Fig. 2A–C).

Reef fishes constituted 38.8% of the entire fauna, with 59.8% of them regional endemics (Table S1). The entire reef fish fauna displayed nearly the same three-cluster arrangement as the whole fauna pattern, with the exception of the inclusion of the southwest Antilles as part of the northern South America cluster (Figs. 2A, 2E, S3D). For endemic reef fishes, the study area was split into four clusters. The main differences between the endemic reef fish and whole fauna configurations were the separation of the SW Gulf of Mexico as a separate cluster most closely linked to the central cluster as well as the inclusion of the Lesser Antilles with the southern cluster for reef fish (Figs. 2E, S3E). Non-endemic reef fishes displayed a tripartite pattern similar to the whole fauna pattern, but with the southern cluster extending along the continental shore of Panama and Costa Rica (Figs. 2F, S3F). For reef fishes, the most divergent fauna in all three cases was that of the northern cluster (Figs. 2D–F, S3D–F). The central cluster had the highest proportion of local species, due largely to its higher proportion of endemics (Fig. 2D–E, Table S1). Proportions of local species were very low in all three clusters for non-endemic reef fishes (Fig. 2F).

Soft bottom fishes represented 38.9% of the fauna, with regional endemics comprising 46% of them (Figs. 3A, B; S4A, B). Both the entire group and endemic and non-endemic components displayed three-cluster configurations very similar to the whole fauna pattern. The only significant exception was the separation from the central cluster of a depauperate endemic-fauna cluster that included part of the north-west Caribbean and Bermuda (Fig. 3B). Among the soft bottom fishes the most divergent fauna in all three cases was that of the northern cluster (Figs. 3A–C, S4A–C). Unlike reef fishes the northern cluster of soft bottom species had the highest proportions of both local and endemic species (Fig. 3A, B; Table S1). Proportions of local species were low in all three clusters for non-endemic soft bottom fishes (Fig. 3C), but higher than compared to non-endemic reef fishes (Table S1).

Pelagic fishes comprised only 19.6% of the fauna, with few of them (17.7%) being regional endemics. Cluster configurations for pelagic species differed from those of reef and soft bottom species by lacking clear northern, central and southern aggregates, and tending towards a north/south split (Fig. 3D–F).

**Figure 4:**
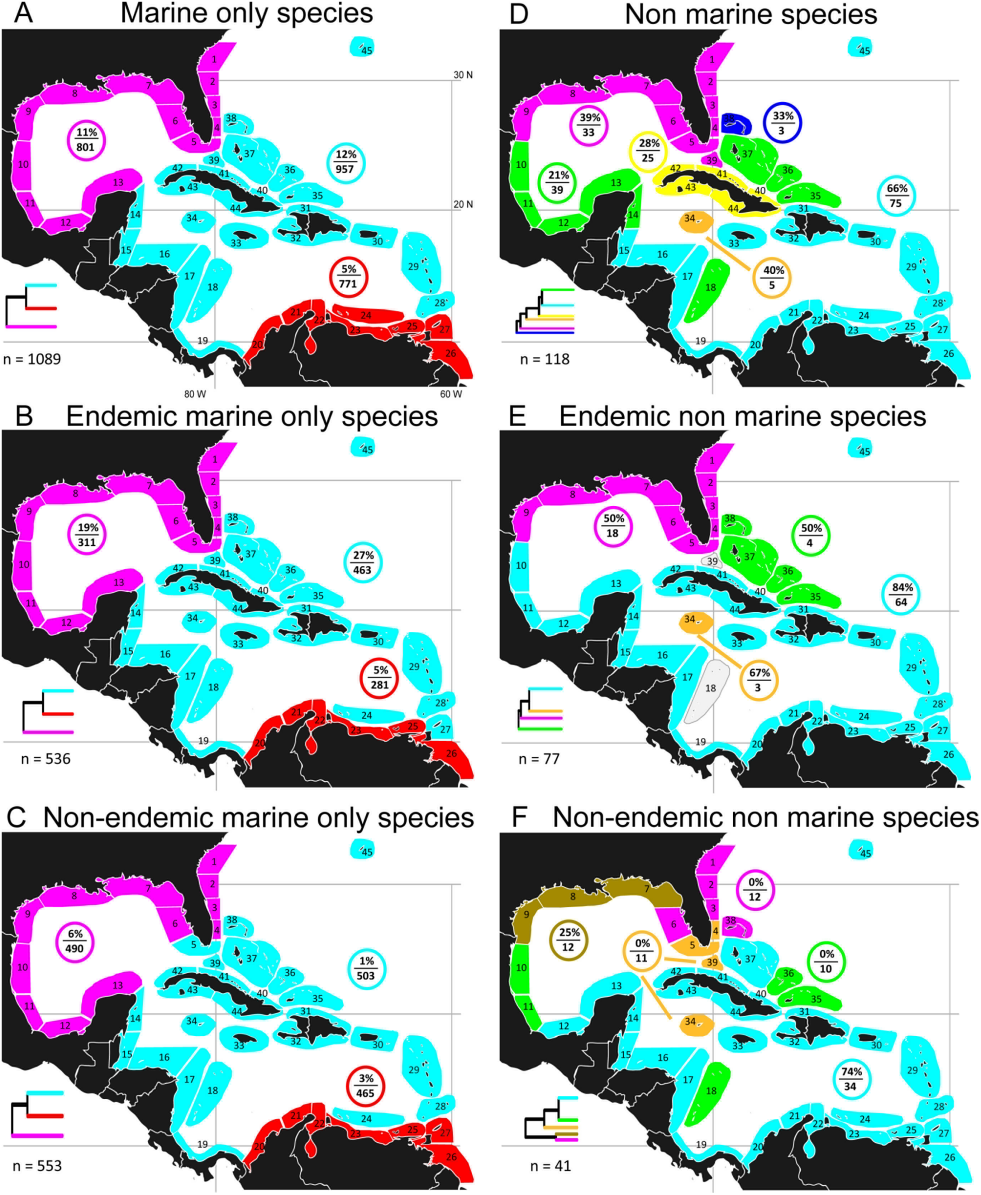
Optimal configuration of major clusters of sites: marine species and non-marine species. Optimal major clusters of sites based on beta-sim dissimilarity dendrograms and evaluation curve fitting (see methods). A–F cluster patterns for subsets of the whole fauna. A–C: all marine species, and their endemic and non-endemic members; D–F: all non-marine species, and their endemic and non-endemic members; %/n in colored circle indicates % of species unique to that cluster and no. species in the cluster; n = total number of species; each dendrogram is a schematic based on the corresponding whole dendrogram in Figure S5, indicating relationships between the major clusters.

Exclusively marine species constituted 69.8% of the fauna, with endemics comprising 49.2% (Fig. 4A–C, Table S1). In contrast, non-marine species represented only 7.6% of the fauna with endemics comprising 65.3% (Fig. 4D–F; Table S1). The cluster patterns of the former paralleled those of the entire fauna (Fig. 4A–C, S5A–C). In contrast, the arrangement for non-marine species was much more fragmented, although with a tendency towards a north/south split (Fig. 4D–F, S5D–F).

Levels of endemism were highest in the central cluster for all species, reef fishes and pelagic fishes, but highest in the northern cluster for soft bottom fishes (Table S1). For all fauna and the various faunal subsets, maximum levels of dissimilarity between clusters were consistently higher for assemblages of regional endemics than for assemblages of non-endemics (Table S1). Maximum levels of dissimilarity within assemblages were greatest for non-marine species, least for pelagic species and higher for soft bottom species than for reef and exclusively marine species (for non-marine species see Fig. S5, for other groups Table S1).

Spalding et al. ([10]; Fig. 1F here) defined 12 subunits (“ecoregions”) spanning our study area, each with a relatively homogeneous fauna “clearly distinct” from the faunas of other ecoregions. The geographic configuration of 12 clusters produced by cutting our dendrogram at 1/3 of the maximum dissimilarity level (see Fig. S3A) was very similar to that of Spalding et al. (compare Figs. 1F and 2A). Petuch [13] defined two provinces and 12 “sub-provinces” within our study area. The boundaries of his Carolinian province are the same as those of the northern province of our whole fauna analysis (Fig. 1H, Fig. 2A). In addition, one of his sub-provinces is equivalent to our whole fauna southern province, another to our Bermuda “ecoregion”, and the rest are generally similar in location to most of our remaining “ecoregions” (albeit with quite different boundaries in many cases–compare Figs. 1H and 2A).

The beta-sim/ANOSIM analysis for the whole fauna produced the same three cluster arrangement (not shown) as the equivalent beta-sim/evaluation curve analysis (see Fig. 2A). Bray-Curtis/ANOSIM produced three main clusters similar to those of beta-sim/evaluation curve, plus a separate Bermuda cluster (Figure S11). All three methods produced the same geographic arrangement for the northern province, and failed to indicate any limits to the Greater Caribbean within the study area. The main differences in the clustering patterns produced by the Bray-Curtis/ANOSIM and beta-sim/evaluation curve methods were that the former produced separate insular and continental-Caribbean provinces and a separate Bermuda province (Fig. S11A). The beta-sim/evaluation curve method also indicated that the Bermuda fauna is one of the most distinctive within the central province (see Figs. 2A and S3, site 45). Because the Bray-Curtis index is susceptible to differences in faunal size as well as composition (see methods), the separation of a Bermuda province by that method (Fig. S11A, C) is likely partly due to Bermuda having the smallest fauna of any of the 45 sites (416 species versus 492–894 species for the remaining 44 sites). Although both Central and South American coasts were included in a single cluster by the Bray-Curtis method, this method identified Central America as a major subdivision of that cluster (Fig. S11C). Similarly, beta-sim identified Central America as a subdivision of the central province, although a relatively minor one (Fig. S11D). The difference in Central American affinities produced by these two methods likely reflects differences in faunal size, as the faunas of the five central American sites (red sites 14–17 and 19 in Fig. S11A) were larger than all but three of 20 insular sites (blue sites in Fig. S11A).

## Discussion

Our analyses show that the study area is occupied by a single biogeographic region, the Greater Caribbean, that divided into three major provinces with distinctive shorefish faunas: a Northern Province that comprises all of the Gulf of Mexico and the southeast USA up to at least 33°N, a Central Province that includes all the offshore islands plus the Central American coast, and a Southern Province that spans the northern coast of South America south to at least 7°N. This pattern differs substantially from most previous biogeographic schemes, which identified a temperate Gulf-centered region and a tropical Caribbean-centered region ([3], [5], [9]–[12]; separate Gulf and Southeast US regions in [4]). Only Ekman [2] and Petuch [13] considered the area occupied by our Northern Province as a subtropical province of the TWA linked to the Caribbean area. All previous analyses underestimated the biogeographic distinctiveness of the fauna of northern South America, which our analysis reveals is a major subdivision of the Greater Caribbean. Despite methodological differences, the fine-scale subdivisions of the study area revealed by our analyses for shorefishes are quite similar to those described by Spalding et al. [10] for shelf organisms in general and somewhat similar to those described by Petuch [13] for certain gastropods. Equivalent analyses to those we describe here with mollusks and other taxa will allow tests of the generality of the patterns we detected with fishes.

### A Northern Province: the Gulf of Mexico and southeastern USA

The patterns produced by our analyses support the view that the entire Gulf of Mexico and southeastern USA represents a single biogeographic unit occupied by a subtropical fauna, i.e., one that is predominantly tropical and contains a minority of temperate elements (see [2]). The great majority (85%) of species present in the northern province are tropical species found in the central and southern zones, and only a small minority (not more than ∼6%) are comprised of temperate species whose ranges extend northwards of the study area. Most previous analyses identified the SW Gulf of Mexico and southern half of Florida as part of the tropical Greater Caribbean, on the basis of their SST regimes and faunas. Our analysis showed that both those areas are faunistically much more closely linked to the remaining parts of the Northern Province than to the Caribbean and West Indies. This linkage of southern Florida, with its large shallow coral reef tract, to the Gulf of Mexico and the rest of the SE USA, was consistent across all different subsets of the fauna (endemics, non-endemics, reef fishes, soft bottom fishes, pelagic fishes, marine species, and non-marine species) with one exception: the few (41 species) of non-endemic, non-marine fishes (Fig. 4F). The southwest Gulf of Mexico also appeared as a consistent part of the Northern Province except for GC endemic reef fishes and its few non-marine species (Fig. 2E, S3E). The southwest Gulf of Mexico and lower Florida areas have tropical faunas and are consistently linked to the remainder of the Northern Province fauna because the provincial fauna is primarily tropical in terms of the numbers and distributions of its component species. Temperate species are widely distributed throughout the northern province (e.g., [8], [26], [27]; Fig. S6 here). These species are concentrated along the entire Atlantic shelf of Florida [8], and extend into the Gulf of Mexico, although some have disjunct distributions that exclude southern Florida [8], [28], [29]. However, such species are not the primary group defining the northern province, as the province exists as a cohesive entity for the primarily tropical regional-endemic species as well as non-endemics.

Surface coastal waters of the northern Gulf of Mexico experience winter SST minima typical of warm temperate regions (∼10°C) and are inhabited by some temperate species [2], [28], [29]. However, a string of small, subsurface coral reefs and rocky pinnacles exists along the edge of the continental shelf of the northern Gulf, although most of the shelf is covered in soft substrata (see Fig. S9A). Live coral formations on the Flower Garden Banks and other shelf-edge pinnacles depend on subsurface winter temperatures that are distinctly higher than those at the surface inshore [2]. These shelf edge pinnacles harbor many tropical reef fishes, and the paucity of reef fish populations in the northern Gulf reflects the tiny amounts of such habitat present in the area as well as effects of winter surface temperatures. An abundance of artificial reef structures (oil rigs) scattered across the northern shelf of the Gulf presents an opportunity to tease out effects of habitat versus temperature on the cross-shelf distributions of tropical and temperate reef fishes in that area. To test if there are general differences in the distributions of species that vary in the degree of their exposure to low winter temperatures of inshore waters of the northern Gulf of Mexico we compared the distribution patterns of species limited to shallow water (20 m or less), and other species that potentially have a refuge from low temperatures in deeper water (lower depth limit exceeds 50 m). Both shallow and deep components of the whole fauna and the regional endemic fauna exhibited the same cluster configurations in relation to the northern Gulf of Mexico (Figure S7). This supports the view that the northern province is a cohesive biogeographic unit not dominated in whole or part by temperate species.

Ekman [2] viewed the warm-water faunal region of the northwest Atlantic as encompassing not only the Caribbean and West Indies but also the Gulf of Mexico and southeast USA up to about 35°N. He proposed that the areas with tropical faunas are bounded by 20°C winter temperatures, while the adjacent zones that experience winter temperatures down to about 16–18°C have subtropical faunas, which are “….thinned out warm-water faunas which have most of their constituents in common with tropical faunas and a much smaller number in common with the neighboring temperate fauna….” and which “….offer a greater contrast with the temperate than with the tropical fauna….and…form a unit with the tropical fauna that will be discussed under the heading “warm-water fauna”” ([2], see p 2). Subsequent major works on the biogeography of the Caribbean area almost invariably have regarded the northern half of the Gulf of Mexico and eastern USA between central Florida and 35°N as having a temperate fauna that is distinct from the warm water TWA fauna ([5], [9], [10]–[12], [28], but see [13]). In contrast, our analysis supports Ekman’s initial view (and see [13]) that this area represents a subtropical province of the warm-water Greater Caribbean, albeit one that does not have a strongly “thinned-out” tropical fauna (Fig. 2). Use of low resolution winter 20°C SST isotherms to define boundaries between temperate and tropical marine biogeographic regions (cf [3], [5], [11], [18]) generates an oversimplified view of the biogeographic significance of temperature conditions on the shelves of the Gulf of Mexico and the southeast USA, where seasonal temperature regimes vary greatly in relation to depth and proximity to shore. Assessment of major and minor faunal boundaries based on quantification of faunal characteristics helps clarify situations such as these, as tropical organisms vary in their susceptibility to temperature stress, many tolerate significantly lower temperatures than 20°C (e.g., [30]), and their distributions are affected by other environmental variables (e.g., reef habitat availability in the northern Gulf of Mexico) in addition to temperature.

Various authors have regarded the Gulf and Atlantic sections of most of our northern province as separate biogeographic regions [4] or provinces [10], [12]. Separation of those two areas is supported by the few studies available that show strong genetic breaks between Gulf of Mexico and south eastern USA populations of conspecifics of various taxa, including fishes (e.g., [31]) and marine invertebrates (e.g., [32], and papers cited therein). The three “ecoregion” configuration of this area in our whole fauna analysis (Fig. 2A) is consistent with that assertion. However, in our analyses, the levels of dissimilarity between the Gulf and Atlantic “ecoregions” of the northern province are much the same as the levels separating other contiguous “ecoregions” within any single province (Fig. S3), indicating that the Gulf/Atlantic separation is not particularly strong. Determining whether there are more substantial differences between the Gulf and Atlantic coast faunas would require a general, multi-taxon analysis that uses genetic and morphological data to reassesses the taxonomic status of “conspecific” organisms on the Gulf and Atlantic coasts, including both provincial endemics and regional non-endemics.

All but one of the biogeographic schemes illustrated in Fig. 1 regarded the warm, southern tip of Florida as a part of what represents the Central Province here. The exception [13] included it as part of what is equivalent to our Northern Province, basing that subtropical biogeographic unit on the occurrence a suite of shared endemic genera and species of mollusks. The inclusion of the tip of Florida as part of a tropical biogeographic unit by other authors largely reflects the conspicuousness and importance placed on the large tract of coral reefs in the warm waters of the Florida Keys. A recent similarity analysis (using betasim/ANOSIM; [18]) of western Atlantic tropical reef fish assemblages also linked the tip of Florida to the rest of the Caribbean. In contrast, our analysis tied that south Florida fauna to the fauna of the remainder of Florida and the Gulf of Mexico, for reef fishes as well as the entire fauna and soft bottom fishes. Several factors likely contribute to that difference in assignment of south Florida in these two similarity analyses: First, our database incorporated data from a broader range of sources and covered more sites throughout Florida than did Kulbicki et al. [18], who based their analysis on published check-lists for the “Florida Keys”. In addition, Kulbicki et al. did not include any other Gulf of Mexico sites in their analysis, included data from a smaller range of sites within the Caribbean than our analysis, and, unlike the present case, did not include elasmobranchs. Finally, there may well be differences in the suites of species of reef-fishes included in the two analyses. Kulbicki et al probably did not detect the distinctiveness of the Southern Province because they included only one site from the area: Tobago island at the extreme eastern edge of the province, and lacked any continental shelf sites between Tobago and Belize.

### A Central Province: the West Indies, Bermuda and Central America

Our analysis shows that the center of the study area is occupied by a tropical province that includes the continental coast of Central America and all the offshore islands: the West Indies (the Bahamas and Antilles (but not the continental islands of Trinidad and Tobago)), the southwest Caribbean atolls, and Bermuda. Further, continental versus insular differences in faunal composition within the central province are relatively minor, although there are numerical differences, with continental sites tending to have more species. This linkage of continental and insular areas of the Caribbean in a single unit is consistent with previous studies that concluded there was little evidence of biologically significant differences between the faunas of those two areas [3], [11].

### A Southern Province: Northern South America

None of the previous large-scale (global or American) studies of biogeographic regionalism of the tropical western Atlantic fauna identified a major subdivision resembling the southern province detected by our analyses. This province was delineated (with modifications) in the whole fauna analyses and most ecological faunal subsets, particularly in the two demersal species subsets (reef and soft bottom). It is also evident in both endemic and non-endemic components of the fauna. The exception was for non-marine species, a minor component of the GC fauna, for which northern South America (NSA) was linked to the rest of the Caribbean.

Although previous large-scale studies did not identify the NSA fauna as wholly distinct from the rest of the GC, some publications hint at such a pattern. In his multivolume treatment of Venezuelan shorefishes [33]–[37], Cervigon noted many cases of species found in the eastern half of northern South America that were present within either the southwest Antilles or mainland Venezuela, but not both locations [33]–[37]. A plot of the distributions of South American shelf fishes whose ranges extend into the Greater Caribbean shows them concentrated in a zone equivalent to our southern province ([7], [10]; Fig. S6). Diaz [38] compared the distributions of gastropods along nine sections of the continental shelf between Costa Rica and Guyana, plus the southwest Antilles. His analysis identified a distinct coastal zone occupying the eastern 2/3 of northern South America (approximately equivalent to our whole fauna NSA province), and faunal separation of the southwest Antilles from the continent similar to that seen in our analyses [13]. The distinctiveness of the recent gastropod fauna of northern South America from the rest of the Caribbean [13], [38] extends back 20+ million years through the Miocene [39], and has some parallels in the Neogene shorefish fauna of eastern Venezuela [40]. Both Spalding et al. [10] and Petuch [13] recognized that, within the Greater Caribbean area, the area equivalent to our southern province is faunally distinct, but considered it to be only one of 12 relatively minor subdivisions of the larger area. Our analysis expands on the results of these previous studies by demonstrating the existence of a NSA shorefish fauna with a species composition sufficiently distinct from the rest of the GC to warrant provincial status.

Compared to the Northern and Central Provinces, which have provincial endemism rates of 9% and 12.2%, the rate of endemism in NSA is fairly low: 3.4% (Table S1). However, once the southern border of this province is determined, its endemism rate will likely increase as some of the species which we classified as non-endemics will likely will turn out to be southern province endemics. Similarly, the endemism rate of the Northern Province may change when its northern limit is more precisely identified. At present we focus on the overall levels of species restricted to each province, which range from 10.1–15.1%, and happen to coincide with or exceed the 10% minimal-endemism limit often used to define biogeographic units [5], [9], [11].

### Northern and southern limits to the Greater Caribbean

We found no evidence of northern and southern boundaries to the GC between 7°–33°N. The northern boundary between tropical and temperate faunal regions in the TWA has been variously identified as either Cape Canaveral (28.5°N) on the central Florida coast [3], [5], [7], [9], [10]–[12], or further north at Cape Hatteras (∼35°N) [2], [13]. The outer parts of the southeast shelf of the USA are influenced by the warm, northward flowing Gulf Stream subsurface current, while surface inshore waters as far south as ∼Cape Canaveral are much colder during winter. Differing temperature conditions in shallow inshore waters and deeper mid- to outer shelf waters likely affect the distributions of some shallow and deep species [2]. To assess if such an effect could influence major faunal patterns we compared the distributions of shallow species (limited to the upper 20 m) and deep species (extending beyond 50 m), reasoning that any general temperature-related breakpoint around Cape Canaveral would be more likely to appear in shallow species. Cluster analysis of all shallow and deep species as well as regional endemics in these two groups revealed no such major faunal breakpoint (Fig. S7).

Our results, coupled with those from previous studies, indicate that the Greater Caribbean likely extends to ∼35°N for shorefishes as well as for gastropods. Mid-twentieth century shorefish literature [29], [44] indicates that many tropical species already extended northwards of that area, as does information on gastropods published near the end of that century [45]. An analysis of the distributions of shorefishes that also occur further north in the eastern USA (Fig. S6A) shows an abundance of such species along the entire southeastern coast from 35°N to the tip of Florida, although with a decline south of Cape Canaveral. Because the temperature regimes of the shelf of the southeast US are three dimensionally complex and spatially and seasonally variable, a clearcut general faunal breakpoint may not exist for shorefishes around Cape Canaveral. Roy *et al.* [45] examined the latitudinal distributions of West Atlantic gastropods and found a gradual northward decline in diversity from 25°–35°N, as well as a strong, sharp peak in the abundance of the end points of species ranges at 35°N, where the Gulf Stream abruptly turns offshore towards Europe (Fig. S13). No equivalent peak in range end points was found at the latitude of Cape Canaveral [45]. Thus, our results are consistent the idea of a general northern boundary to the TWA near Cape Hatteras.

Could recent anthropogenic sea warming have shifted the northern limits of shorefish geographic ranges on the eastern US shelf sufficiently to have obliterated a previous regional boundary for the GC at central eastern Florida? Changes in the distributions of shorefishes on the US shelf north of Cape Hatteras (between 36°–45°N) have occurred during the last 50 y as sea temperatures have gradually increased [41]. These include range expansions (10 of 36 species), northward shifts in the centers of biomass (17 species), northward extensions of northern range limits (five subtropical species), and a northward retreat of the southern range limit of one temperate species. Two other studies have also found changes in the distributions of northern Gulf of Mexico fishes coincident with sea warming since the 1970–80s. In one case involving fishes living in shallow seagrass beds in the northeastern Gulf ([42]; note that data from this study were not included in our database), two subtropical and 14 widespread (tropical and subtropical) shorefish species present in 2006/2007 were absent the 1970s. This is consistent with a northward shift of as much as 3° of latitude in the northern range limit of some species into a shoreline zone that has relatively cold winter SSTs and that has warmed markedly (>3°C; [42]) during that period. However, interpretation of these changes is complicated by the loss from that study area during the same period of 19 widespread and 10 subtropical species. In addition it is unclear whether these changes were related to natural decadal-scale climate fluctuations rather than a longer term anthropogenic warming trend [42]. A second study [43] found that fishes in the central shelf of the northern gulf have shifted the centers of their local distributions away from the coast towards the shelf edge since the late 1980s. Thus long-term changes in fish distributions associated with sea warming can be complicated and variable. Testing for range-limit changes requires intensive, long-term repetitive sampling over large spatial scales, as exemplified by those three studies. We know of no similar analyses of changes in the ranges of tropical shorefishes in the southeastern US that might have occurred in sufficient numbers of species to have affected any general faunal boundary around Cape Canaveral.

There is no previous general consensus about the southern limit to the Greater Caribbean, which has ranged from the Gulf of Paria, between Trinidad and Venezuela, to Suriname [13], to various points scattered throughout the length of Brazil (see Fig. 1, and [7]). Roy *et al.* [45] found a small peak in the abundance of gastropod range end-points at the latitude of the north coast of South America. The lack of consensus reflects the co-occurrence of many common taxa in the Caribbean and Brazil, the lack of any abrupt SST changes connected with faunal changes in the boundary area, the lack of comprehensive information on faunal distributions along the north-east coast of South America, and a lack of quantitative analyses relating to this question. Extensive areas of hard bottoms that support sponges and corals along much of the edge of the shelf between the Orinoco and Amazon rivers harbor tropical reef fishes and other benthic reef organisms found in both the Caribbean and Brazil [46]–[48], blurring any boundary between those areas. Data on the distributions of species that are found in both the Caribbean and further south [7] indicate a breakpoint within the Caribbean at northeastern Venezuela (in segment 25 of Fig. 1; see Fig. S6B, and also [7]). However, our analyses do not support the notion of a major breakpoint there or as far south as northern Guyana. Quantitative analysis of shorefish distributions along the entire tropical eastern coast of South America are needed to resolve the location of the southern limit for the Greater Caribbean and the biogeographic relationship between the shorefish faunas of the Southern Province of the GC and Brazil.

### The Three-province configuration as a product of environmental variation

Understanding the roles of historical and modern environmental forces in shaping contemporary biogeographic patterns is a central part of marine biogeography [2], [4], [5], [13], [20], [41], [49], [50]. In our study of provincial subdivisions of the TEP we concluded that a lack of barriers to pelagic dispersal between provinces and evidence of recent speciation within them implicated environmental differences between provinces as the driver of faunal differences [19]. Because the Greater Caribbean area is geographically much more complex than the TEP there could be greater limitations on connectivity between provinces than exists with the TEP. However, two recent events clearly demonstrate that there are no general limitations on connectivity between the three GC provinces. First, pathogen-produced mass mortality of the sea urchin *Diadema antillarum* spread from Panama throughout the three provinces of the Greater Caribbean during 1983–84 [51]. Second, the introduced Indo-Pacific lionfish (*Pterois* spp.), which produces pelagic larvae, spread explosively from its origin at the southeast tip of Florida throughout the entire region over the past decade, both northwards, with the prevailing surface current flow, and southwards against that flow ([52]; animation of lionfish spread to date at http://nas.er.usgs.gov//queries/SpeciesAnimatedMap.aspx?speciesID=963; for surface currents see Fig. S13, and animation at http://marinebio.org/oceans/currents-tides.asp). Changing sea levels and temperatures during Pleistocene glacial periods likely had some localized effects on connectivity within parts of provinces (e.g., within the Bahamas– e.g. [53]; and between the two coasts of Florida– e.g. [29]). However, sea level changes did not produce additional barriers between the three provinces because they are connected by deep water. Recent genetic evidence is consistent with speciation in response to differences in ecological conditions between Greater Caribbean provinces in the face of dispersal across provincial boundaries and within TWA regions for species known to disperse between regions [54]–[56].

Reef and soft bottom fishes, the two most important ecological groups in the Greater Caribbean regional fauna (together 77.6% of all species) demonstrated the same three-province arrangement of the study area. In contrast, among pelagic fishes the tendency was towards a bipartite north-south division. This indicates that both groups of demersal fishes, the main drivers of the whole-fauna pattern, are responding to the same general set of differing provincial environmental conditions on the continental and insular shelves (cf [7]).

#### The Northern Province: a heterogeneous, largely subtropical and eutrophic environment

Conditions in much of the Northern Province are subtropical (15–20°C winter lows for SST), with temperate winter conditions (<15°C) close inshore along the northern coast of the Gulf of Mexico (Ekman 1953) and the southeastern US coast northwards from Cape Canaveral. The southern Gulf of Mexico and southern Florida are more tropical although conditions are cooler there than anywhere else in the study area except the northern Bahamas and Bermuda (Fig. S8A). As the latter two sites are dominated by coral reefs and belong to a different faunal province, SST alone is not decisive in defining the finer scale boundaries of either the northern or central provinces, even though it may be a good general predictor of biogeographic boundaries at the global scale [20]. A medium sized seasonal upwelling affects the northern coast of the Yucatan Peninsula ([57], [58], and Fig. S8A, B). There apparently is minor upwelling activity along parts of the western and northwest Gulf of Mexico [59], as well as upwelling associated with the flow of the Gulf Stream along the edge of the southeastern USA shelf north of Cape Canaveral [60], [61]. Much of the Gulf of Mexico has substantial freshwater and nutrient outflows from large rivers entering along the western and northern shores, moderately large rivers in the southwest, and the Everglades wetlands in the southeast (Fig. S8B). Thus near-shore conditions in much of the northern province generally are colder (in winter) and more eutrophic than those in the relatively oligotrophic Central Province.

The shoreline of the Northern Province is almost entirely soft bottom, bounded by extensive coastal wetlands and lagoon systems with varying salinities, and an abundance of mangroves in the southwest Gulf of Mexico and southern Florida (Figure S9C, D). However, a wide continental shelf allows development of offshore coral reef habitats away from immediate coastal influences. There are scattered emergent coral reefs in the southwest Gulf of Mexico, a string of small submerged coral reefs and pinnacles along the outer edge of the northern shelf of the Gulf, and extensive areas of low relief, rocky “hard-bottom” that support reef fishes on the vast, shallow west Florida shelf (Fig. S9A, and see [29]). The Florida keys have a large coral reef system, and extensive “hard-bottom” areas and deep coral reefs occur all along the central and outer parts of the shelf of the southeastern USA (Fig. S9A). The Northern Province, with large areas of diverse tropical and subtropical habitats, as well as inshore areas of more temperate habitat, supports a fauna only a little smaller than that of the Central Province (Table S1). This fauna includes a relatively large number of local endemics (9%) as well as regional non-endemics (6.1%) that have temperate affinities and also occur northwards along the US coast. It is further distinguished by having the lowest percentage of reef fishes, and the lowest ratio of reef species to soft bottom species in its fauna (Table S1), a reflection of its huge area of soft bottom shelf.

#### The Central Province: a tropical, mainly oligotrophic area rich in coral reefs

Environmental conditions on the shelves of the islands and Central America are very different from those on the continental shelves of the other two Provinces. Due to the narrowness of the Central American Isthmus and position of its central mountain range there are no large rivers delivering freshwater to its Caribbean shelf (Fig. S8B). The two continental areas of high rainfall with medium sized rivers (Nicaragua and Guatemala/Belize) (Fig. S9A, B), do have soft bottom coasts with extensive mangroves (Fig. S10C, D). However, substantial areas of coral reef are present along the Central American coast, including Mexico, the outer edge of the narrow shelf at Belize, the central parts of the shelf off Honduras and Nicaragua, and near the edge of the narrow shelf off Panama (Fig. S10A). The offshore islands also possess an abundance of coral reefs (Fig. S10A). Unlike the other two provinces the Central Province lacks sustained upwelling systems of significant size. Thus, in contrast to the situation on the continental shelves of the other two provinces, large areas of the Central American continental shelf have oligotrophic conditions and habitats like those of the islands. Restrictions on the development of inshore reefs imposed by soft bottom coastlines and river-runoff on the coasts of Honduras and Nicaragua are offset by a wide continental shelf on which offshore reefs support reef fish assemblages. This tropical, coral-reef rich Central Province, which has the largest area of the three provinces, contains the highest number of species, the highest percentage of local endemics in its fauna (12.2%) and the highest ratio of reef species to soft bottom species (Table S1).

#### The Southern Province: a eutrophic, upwelling-affected area marginal for coral reef development

Both the historical and current distinctiveness of the southern province marine faunas have been linked to environmental conditions peculiar to that area [13], [38]. These include high nutrient inputs from coastal upwelling systems scattered along the eastern 2/3 of that region ([28], [62], and see Fig. S8A, B here), and from outflows from large rivers that drain high rainfall areas at both ends and the center of that area ([62], and Figs. S9A, B & S8B). Upwelling conditions not only affect shelf productivity, but also stress tropical organisms with low temperatures and reduced pH [63]. Current and historical eutrophic conditions produced by upwellings are similar in both the TEP and NAS [13], [38], [39], [50]. As with other upwelling-affected, marginal areas in the tropics [64] structural coral reefs are relatively uncommon along the NSA coast, where rocky substrata provide most reef fish habitat (Fig. S9A, B). The largest areas of soft shoreline and soft bottom on the NSA shelf are reduced-salinity areas at the mouths of Lake Maracaibo and the Orinoco River (Fig. S9C). This combination of extensive upwelling systems, large river outflow, and predominantly rocky shorelines is not seen elsewhere in the Greater Caribbean. Among the three provinces, the southern province has the smallest amount of habitat, the lowest number of species, and the lowest percentage of local endemics (3.4%), but the highest percentages of local non-endemics (6.7%), reef fishes and soft bottom fishes in its fauna (Table S1).

Belanger *et al.* [20] used a cluster analysis of local variation in SST, salinity and productivity to define a global set of shelf oceanographic units. Their results for the Caribbean area (Fig. S12 here) reflect environmental differences noted above between the three biogeographic provinces discerned by our analysis: the northern-province area is environmentally the most divergent and spatially heterogeneous of the three, while the central-province area (both central America and the islands) is the most uniform (except for the northern Bahamas and Bermuda – see comments above about SST). These environmental differences are paralleled in the results of our whole fauna analysis, which show faunal dissimilarity being greatest between the northern and other two provinces, and least between the central and southern provinces, reinforcing conclusions about the relationship of the three-province faunal scheme to marked large-scale environmental variation.

### The Greater Caribbean and Tropical Eastern Pacific: a comparison of sister regions

The TEP and GC are sister biogeographic regions with a common faunal heritage that diverged as the Central American isthmus rose and finally closed ∼3 mya [65]. Not only do their shorefish faunas show strong taxonomic similarities due to this history, but both regions show similarities in their internal biogeographic structure, with a warm-water fauna split into three distinctive provincial faunas [19].

Strong regional endemism is the most important defining factor for the TEP shorefish fauna and a major factor for the GC fauna. Although the geography of local endemism contributes to provincial subdivisions of both regions, it is of greatest importance at the regional scale. A range of responses by different components of each provincial fauna to marked geographic variation in general environmental conditions within each region have produced taxonomically and ecologically distinctive provincial faunas.

Each region includes a northern subtropical province centered on a large gulf in which winter SST conditions range from subtropical in the south to temperate inshore in the extreme north. In both regions this province supports a distinctive fauna that consists primarily of species that also occur in more tropical parts of the region, but also includes a particular mix of ecological groups, local endemics and a small minority of temperate species that are also found in an adjacent northern temperate area.

Each region includes a eutrophic continental province with an abundance of upwelling areas that is a marginal environment for coral reef development. In the TEP this province is the largest in terms of habitat area and occupies all the continental shelf outside the northern (Gulf of California) province. Its fauna includes a broad mixture of ecological groups, with many local endemics. In the GC the equivalent province is the smallest and also continental, and has a relatively small fauna defined by: a distinctive mix of ecological groups, species shared with areas further south along the south American coast, and a small proportion of local endemics.

Both regions have a province centered on islands. In the GC this includes an abundance of islands of greatly varying sizes plus a substantial but smaller area central section of the mainland. This Central Province is characterized by an oligotrophic environment that has promoted the development of an abundance of structural coral reefs, but which lacks strong isolation among islands and between islands and the mainland. In the TEP, however, the equivalent province is exclusively insular and comprises a few, relatively small, isolated oceanic islands with strong inter-island variation in environmental conditions. The TEP Insular Province has a distinctive fauna defined by a combination of an abundance of single and multi-island endemics produced by strong island isolation, a paucity of ecological groups due to limited habitat diversity, and disproportionately large numbers of regional non-endemics (transpacific species that also occur on central and west Pacific islands; [18], [19]). In the GC the equivalent, Central Province is distinguished almost entirely by its compliment of provincial endemics, as it has very few GC non-endemics that are not found in either or both the other two provinces. While structural coral reefs are rare in the TEP (∼20 km^2^ total in the region), in the Greater Caribbean faunal distinctiveness of the Central Province is related to habitat conditions that include an abundance of coral reefs (∼20,000 km^2^ in the region).

The biogeography of the shorefish faunas of the TEP and GC differ in two other ways. First, the TEP is much more physically isolated from other tropical areas compared to the Greater Caribbean, which has good faunal connections to Brazil. As a result non-endemics form a much larger component of the GC (∼55%) than TEP fauna (∼20%). Second, the regional limits of the TEP are strongly constrained and clearly defined by relatively abrupt changes in temperate conditions at its northern and southern edges, where equator-bound cold boundary currents turn westward away from the continental coast. In contrast there is a northerly flow of warm water into, through and out of the GC (Fig. S13; and for an animation see http://marinebio.org/oceans/currents-tides.asp). This enhances connectivity between Brazilian and GC reef areas, and is largely responsible for less well defined biogeographic boundaries in the Greater Caribbean.

### Some management implications and the biogeographic significance of ongoing taxonomic research

Our results have two, fairly general management implications: First, currently available information indicates that, as is generally thought, most species of shorefishes are widespread throughout much of the GC. The lack of large-scale impediments to connectivity via pelagic larval dispersal between different parts of the region contributes to that situation. Major variation in shelf environments is responsible for substantial differences in the faunas of the three provinces of the GC. Tropical upwelling systems produce distinctive biotas, and identification of northern South America as a major subdivision of the GC with a distinctive fauna linked to its marginal environment indicates that management measures within that area might benefit from reassessment and coordination. Second, our analysis provides strong support for the previous subdivision of the GC into 12 small ecoregions (cf [10]), each with special faunal and environmental characteristics. The combination of an enlarged GC region that contains 12 small ecoregions within three large provinces provides a perspective for both regional and local scale planning to ensure effective management of the entire regional biota.

Are the results of the present analysis likely to be definitive in terms of clarifying the limits and major subdivisions of the GC, and might future research on shorefishes change the patterns indicated by our results? Currently the northern and southern limits of the GC are far from clear. Only detailed information relating to shorefish distributions to the north (beyond 35°N) and south (throughout Brazil) of our study area will clarify that situation. Elucidation of the biodiversity of shorefishes in the TWA in general and GC in particular is far from complete. The accumulation of newly described species has continued steadily over the past 100 years and, particularly among regional endemics, shows no signs of any recent slowdown (Fig. S14). The surge in recent taxonomic studies on GC shorefishes that have been enhanced by the addition of a major new taxonomic tool, forensic barcoding, clearly demonstrates that a major revision of species geography is in the making in the GC and other parts of the TWA. In many cases what was previously thought to be a single species that was widely distributed throughout the TWA has fragmented into multiple genetically and morphologically distinct species. In some instances this has led to a split into a northern species based on the Northern Province, and a southern species based on the Central Province, and further south (e.g., [66], [67]). In other cases species widespread within the Central and Southern Provinces have fragmented into numerous local insular and continental endemics (e.g., [68], [69]). In addition, recent taxonomic reassessment of Brazilian populations of species thought occur both there and in the GC has revealed them to be Brazilian endemics (e.g. [70]). Future reassessments of supposedly conspecific populations in different GC provinces and in the Gulf of Mexico versus the Atlantic coast of the US inevitably will result in further similar splitting. How such revisionary taxonomy will eventually affect large and small scale biogeographic patterns among GC shorefishes cannot be predicted, as so few taxa have been examined to date. Clarification of the intraregional biogeography of areas such as the GC, which is necessary for effective management of the regional biota, seems unlikely to occur very soon, due to a combination of an general, long-term decline in support for taxonomy as a discipline (e.g. [71]) and a growing antipathy towards small-scale collecting for taxonomic and biogeographic studies (e.g. [72], [73]).

## Supporting Information

Figure S1 **Distribution of species occurrence records in the study area.** Combined plot of georeferenced site records for all species used in the construction of the detailed species range maps included in the analyses. Sources of records in Appendix S1. Note: this figure also includes (a small number of) records for non-resident species, which were not used in the analyses.
(TIF)

Figure S2 **Example evaluation curve used to determine major cluster configurations.** Evaluation curve demonstrating L method for finding the inflexion point of the curve (see methods) to establish optimal number of major clusters for the whole fauna assemblage. Local species = species found only in a particular cluster.
(TIF)

Figure S3 **Hierarchical cluster dendrogram of beta-sim dissimilarities between the 45 site faunas: all species and all reef fishes.** A–C: all species, all endemic species, all non-endemic species; D–F: all reef fishes, endemic reef fishes, non-endemic reef fishes.
(TIF)

Figure S4 **Hierarchical cluster dendrogram of beta-sim dissimilarities between the 45 site faunas: soft bottom and pelagic fishes.** A–C: all soft bottom species, endemic soft bottom species, non-endemic soft bottom species; D–F: all pelagic fishes, endemic pelagic fishes, non-endemic pelagic fishes.
(TIF)

Figure S5 **Hierarchical cluster dendrogram of beta-sim dissimilarities between the 45 site faunas: marine and non-marine fishes.** A–C: all marine species, endemic marine species, non-endemic marine species; D–F: all non-marine species, endemic non-marine species, non-endemic non-marine species.
(TIF)

Figure S6 **Distribution of shelf fishes also found in areas to the north and south of the Greater Caribbean.** A: Species found further north, B: species found further south; Figs. 14 and 15 of ref (1).
(TIF)

Figure S7 **Major cluster configurations for shallow and deep species.** Optimal configuration of major clusters of sites based on beta-sim dissimilarity dendrograms and defined by evaluation curve fitting (see methods). A: Species restricted to 20 m depth or shallower; B species whose depth ranges extend below 50 m. C & D endemic subsets of A & B respectively. %/n in colored circle indicates % of species unique to that cluster and no. species in the cluster; each dendrogram is a schematic based on the corresponding whole dendrogram (not shown) that indicates relationships between the major clusters; n below schematic = total number of species.
(TIF)

Figure S8 **Average sea surface temperatures and chlorophyll concentrations in the study area.** A: Average sea surface temperature (July 2002–October 2013), B: chlorophyll concentration (November 2011–October 2013). Source: Aqua MODIS data publically available at http://oceancolor.gsfc.nasa.gov/cgi/l3, accessed 2013 November 27.
(TIF)

Figure S9 **Rainfall patterns and river catchments in the study area.** A: Distribution of rainfall, B: Distribution of river catchments throughout the study area. Images courtesy R. Lammers, Water Systems Analysis Group, University of New Hampshire.
(TIF)

Figure S10 **Habitat types in the study area.** Schematic representations of distributions of different habitat types in the study area. Sources for A, C & D: (2–4); sero.nmfs.noaa.gov/hcd/pdfs/efhdocs/gom_efhhapc_poster. http://ocean.floridamarine.org; inspection of Google Earth images; B: Northern South America coral reef distribution after Maps 5f and 6e of ref (2).
(TIF)

Figure S11 **Major cluster configurations produced by different analytical methods.** Optimal configuration of major clusters of sites in the study area based on dendrograms from Bray-Curtis/ANOSIM and beta-sim/evaluation curve analyses of the whole fauna (see methods). A: Bray-Curtis/ANOSIM cluster pattern, B: Beta-sim/evaluation cluster pattern, C: Bray-Curtis dendrogram, D: beta-sim dendrogram. %/n in colored circle indicates % of species unique to that cluster and no. species in the cluster; each dendrogram is a schematic based on the corresponding whole dendrogram, indicating relationships between the major clusters; n below schematic = total number of species.
(TIF)

Figure S12 **Environmental heterogeneity throughout the study area.** Cells with similar regimes of primary productivity, sea surface temperature and salinity have similar colors, dissimilar cells have dissimilar colors. With permission, from Fig. 1E of (5).
(TIF)

Figure S13 **Surface ocean currents in the study area.** Map courtesy of EH Ryan (eryan@rsmas.miami.edu).
(TIF)

Figure S14 **Accumulation of species descriptions of Greater Caribbean shorefishes.** Accumulation curves and running means of rates of description per year for regional endemics and non-endemics. Source: F Zapata and DR Robertson, unpublished data.
(TIF)

Table S1 **Levels of dissimilarity, unique occurrence and endemism in major site-clusters among different assemblages of shorefishes in the study area.**
(TIF)

Appendix S1 **Sources of data on shorefish distributions used to construct the species range-map database used in the analyses.**
(DOC)
