# Supplementary figures and images for: Defining and Dividing the Greater Caribbean: Insights from the Biogeography of Shorefishes
Source: PLoS One. 2014 Jul 23;9(7):e102918. doi: 10.1371/journal.pone.0102918 (PMC4108436; doi:10.1371/journal.pone.0102918)

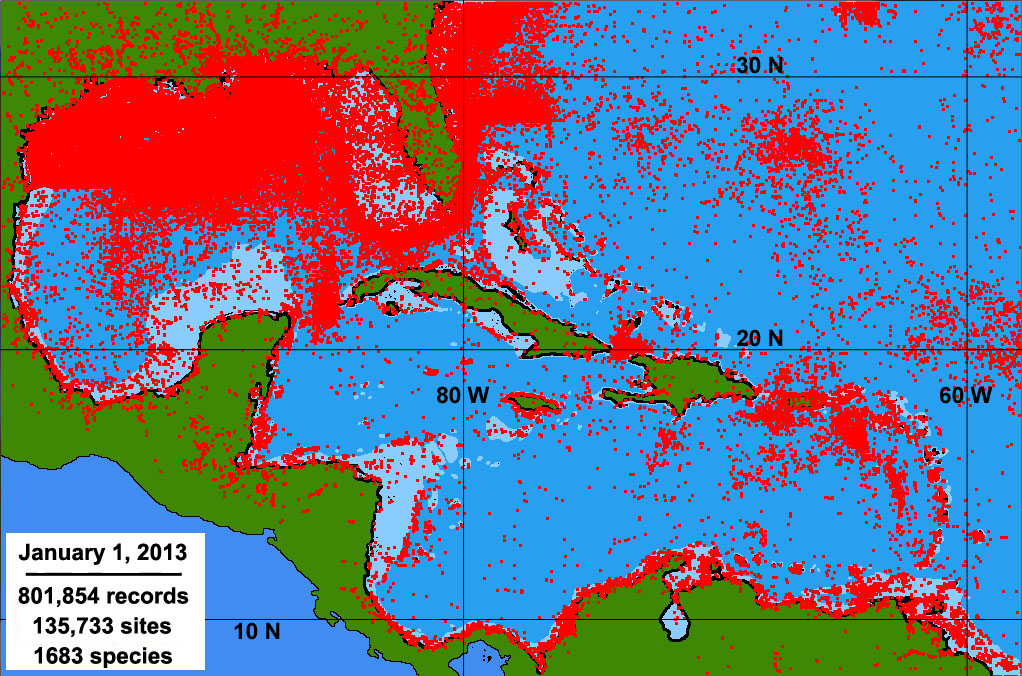

Supplement: Figure S1 — Distribution of species occurrence records in the study area. Combined plot of georeferenced site records for all species used in the construction of the detailed species range maps included in the analyses. Sources of records in Appendix S1. Note: this figure also includes (a small number of) records for non-resident species, which were not used in the analyses. (TIF) [file pone.0102918.s001.tif]

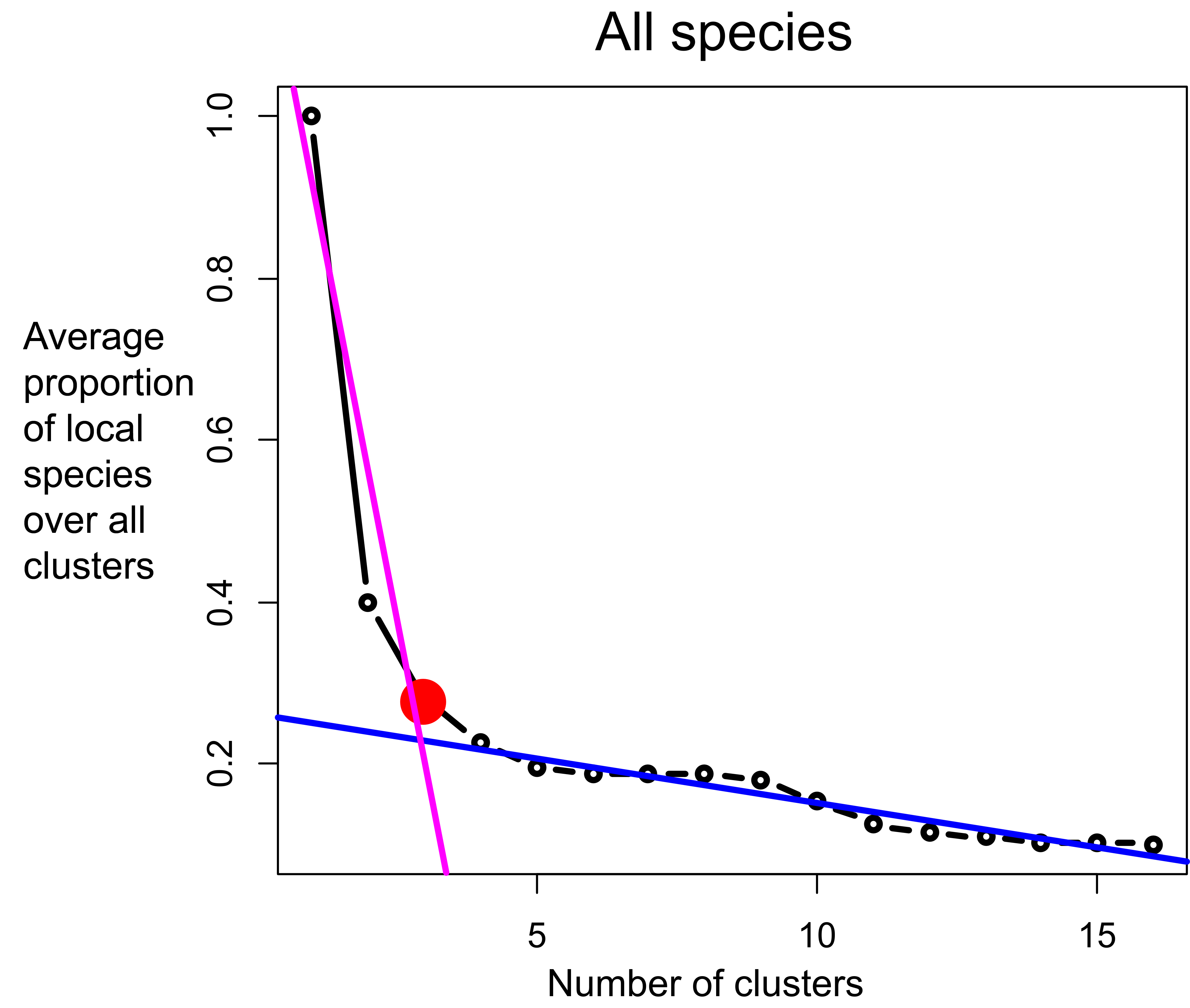

Supplement: Figure S2 — Example evaluation curve used to determine major cluster configurations. Evaluation curve demonstrating L method for finding the inflexion point of the curve (see methods) to establish optimal number of major clusters for the whole fauna assemblage. Local species = species found only in a particular cluster. (TIF) [file pone.0102918.s002.tif]

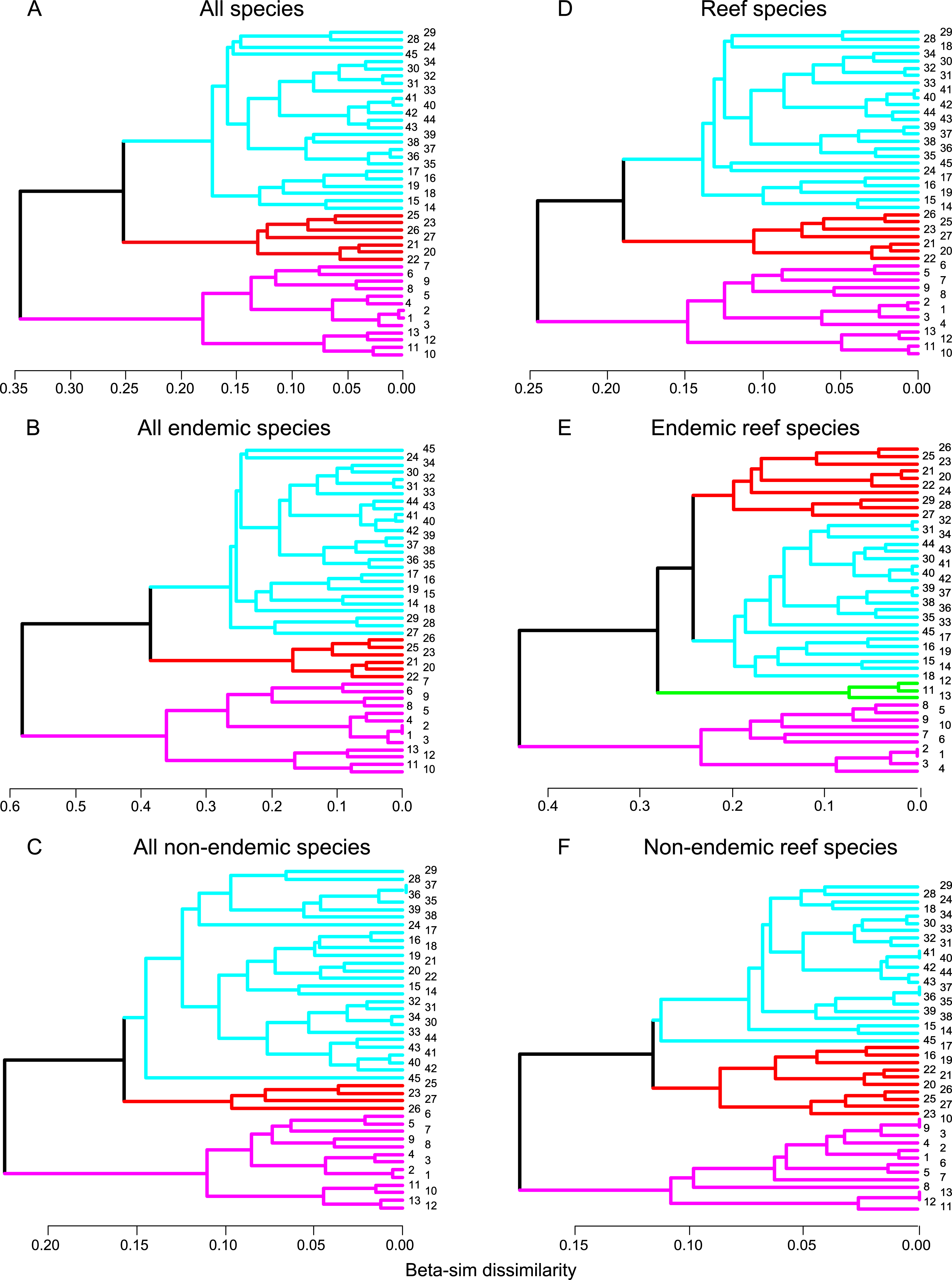

Supplement: Figure S3 — Hierarchical cluster dendrogram of beta-sim dissimilarities between the 45 site faunas: all species and all reef fishes. A–C: all species, all endemic species, all non-endemic species; D–F: all reef fishes, endemic reef fishes, non-endemic reef fishes. (TIF) [file pone.0102918.s003.tif]

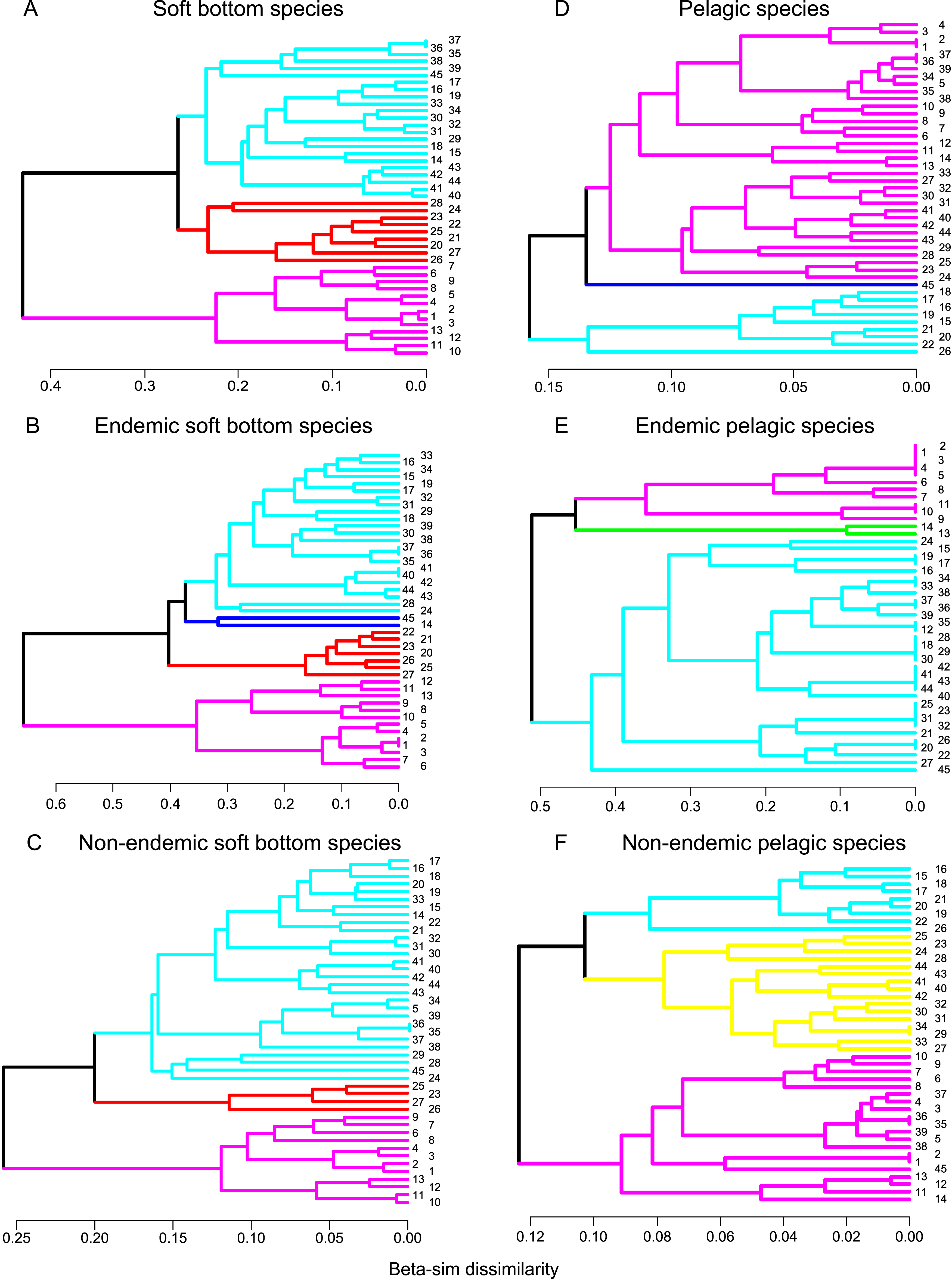

Supplement: Figure S4 — Hierarchical cluster dendrogram of beta-sim dissimilarities between the 45 site faunas: soft bottom and pelagic fishes. A–C: all soft bottom species, endemic soft bottom species, non-endemic soft bottom species; D–F: all pelagic fishes, endemic pelagic fishes, non-endemic pelagic fishes. (TIF) [file pone.0102918.s004.tif]

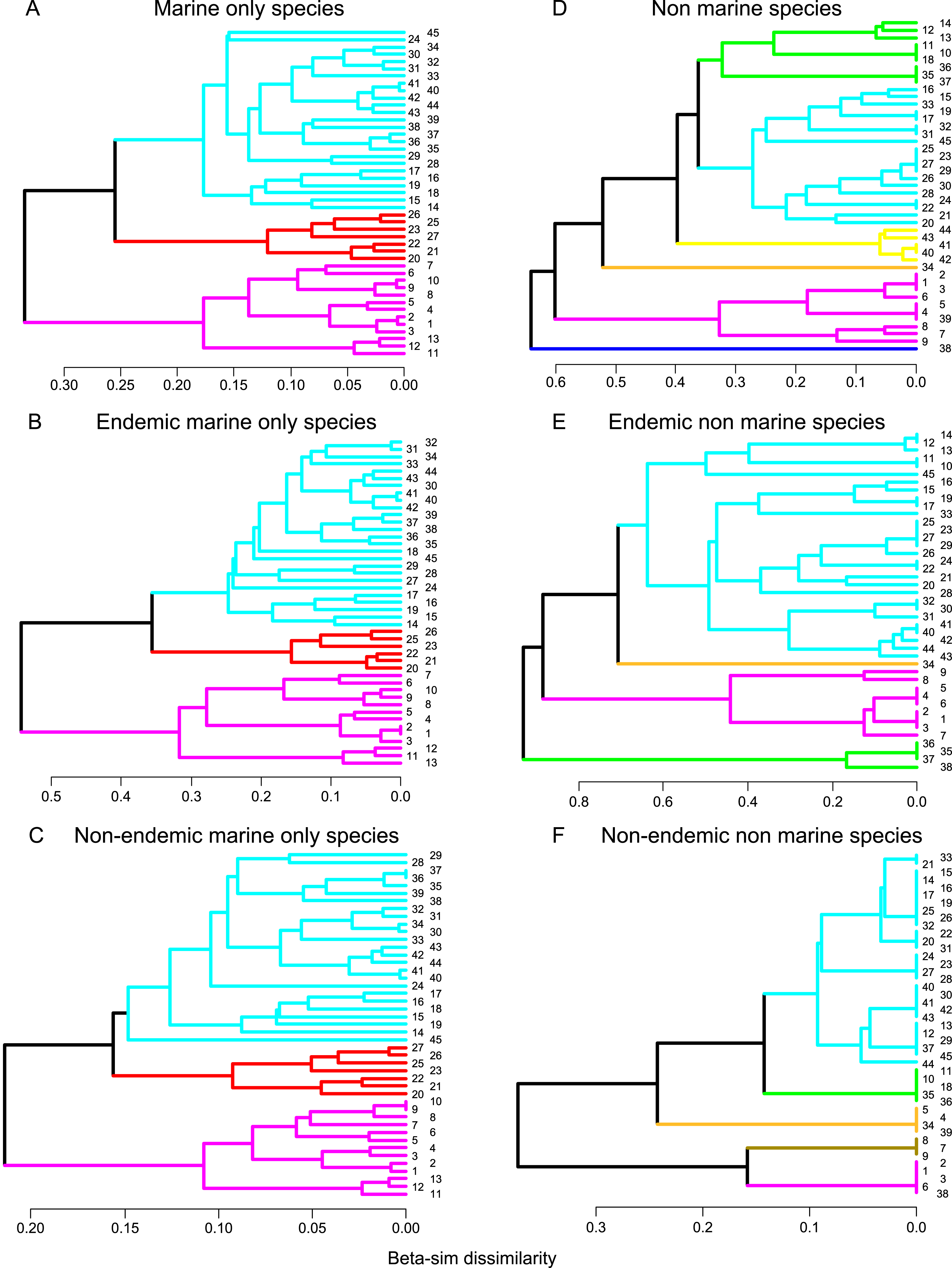

Supplement: Figure S5 — Hierarchical cluster dendrogram of beta-sim dissimilarities between the 45 site faunas: marine and non-marine fishes. A–C: all marine species, endemic marine species, non-endemic marine species; D–F: all non-marine species, endemic non-marine species, non-endemic non-marine species. (TIF) [file pone.0102918.s005.tif]

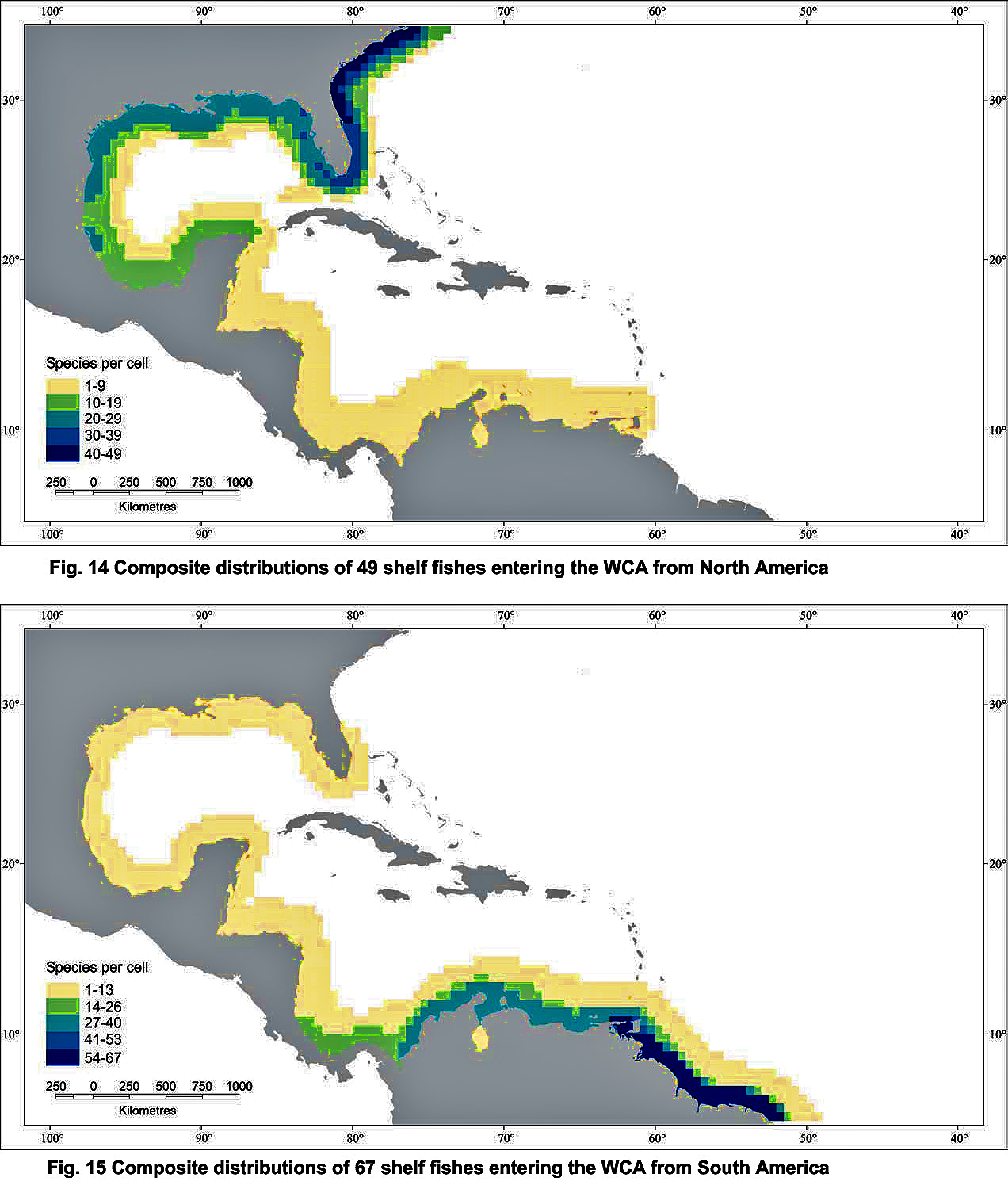

Supplement: Figure S6 — Distribution of shelf fishes also found in areas to the north and south of the Greater Caribbean. A: Species found further north, B: species found further south; Figs. 14 and 15 of ref (1). (TIF) [file pone.0102918.s006.tif]

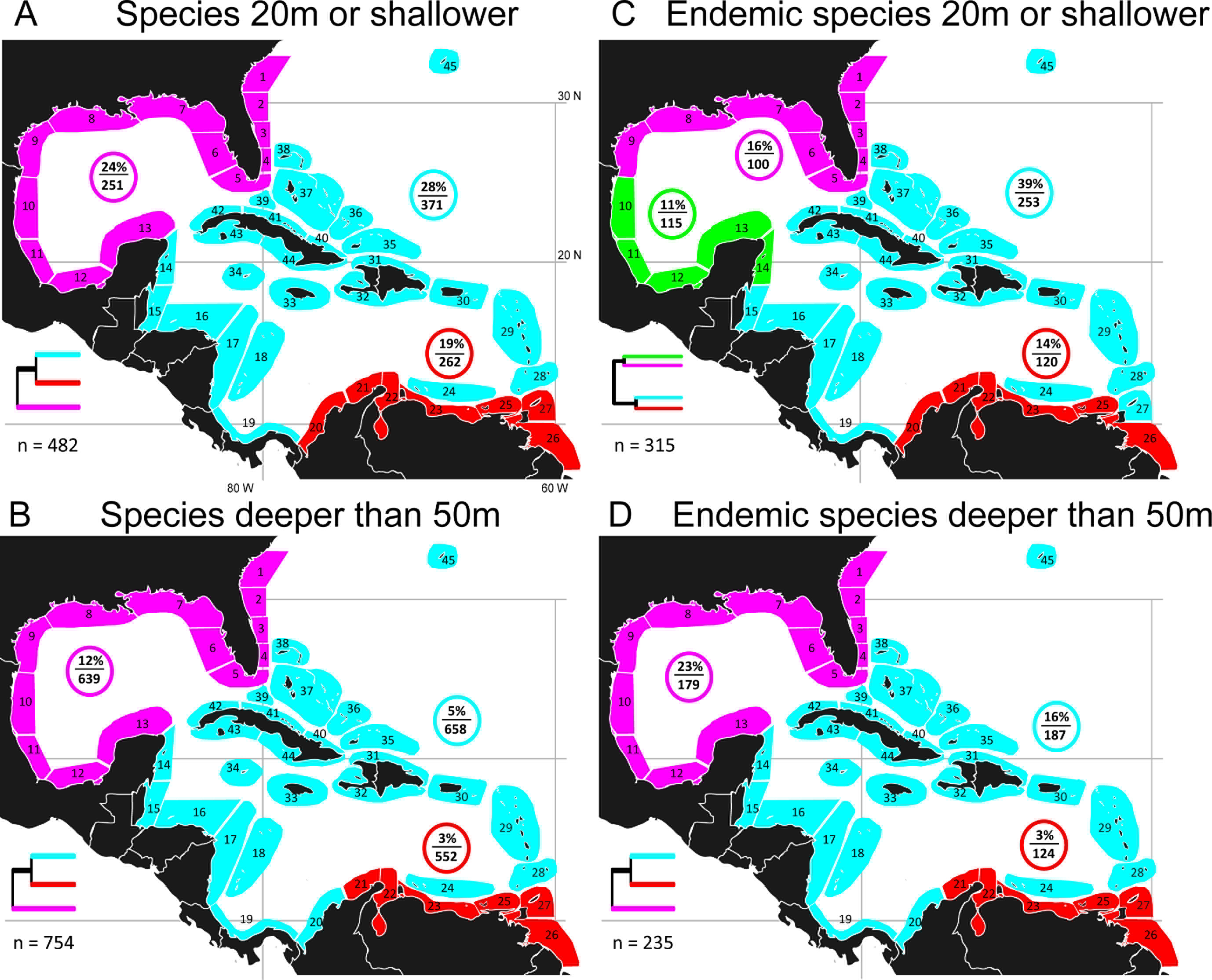

Supplement: Figure S7 — Major cluster configurations for shallow and deep species. Optimal configuration of major clusters of sites based on beta-sim dissimilarity dendrograms and defined by evaluation curve fitting (see methods). A: Species restricted to 20 m depth or shallower; B species whose depth ranges extend below 50 m. C & D endemic subsets of A & B respectively. %/n in colored circle indicates % of species unique to that cluster and no. species in the cluster; each dendrogram is a schematic based on the corresponding whole dendrogram (not shown) that indicates relationships between the major clusters; n below schematic = total number of species. (TIF) [file pone.0102918.s007.tif]

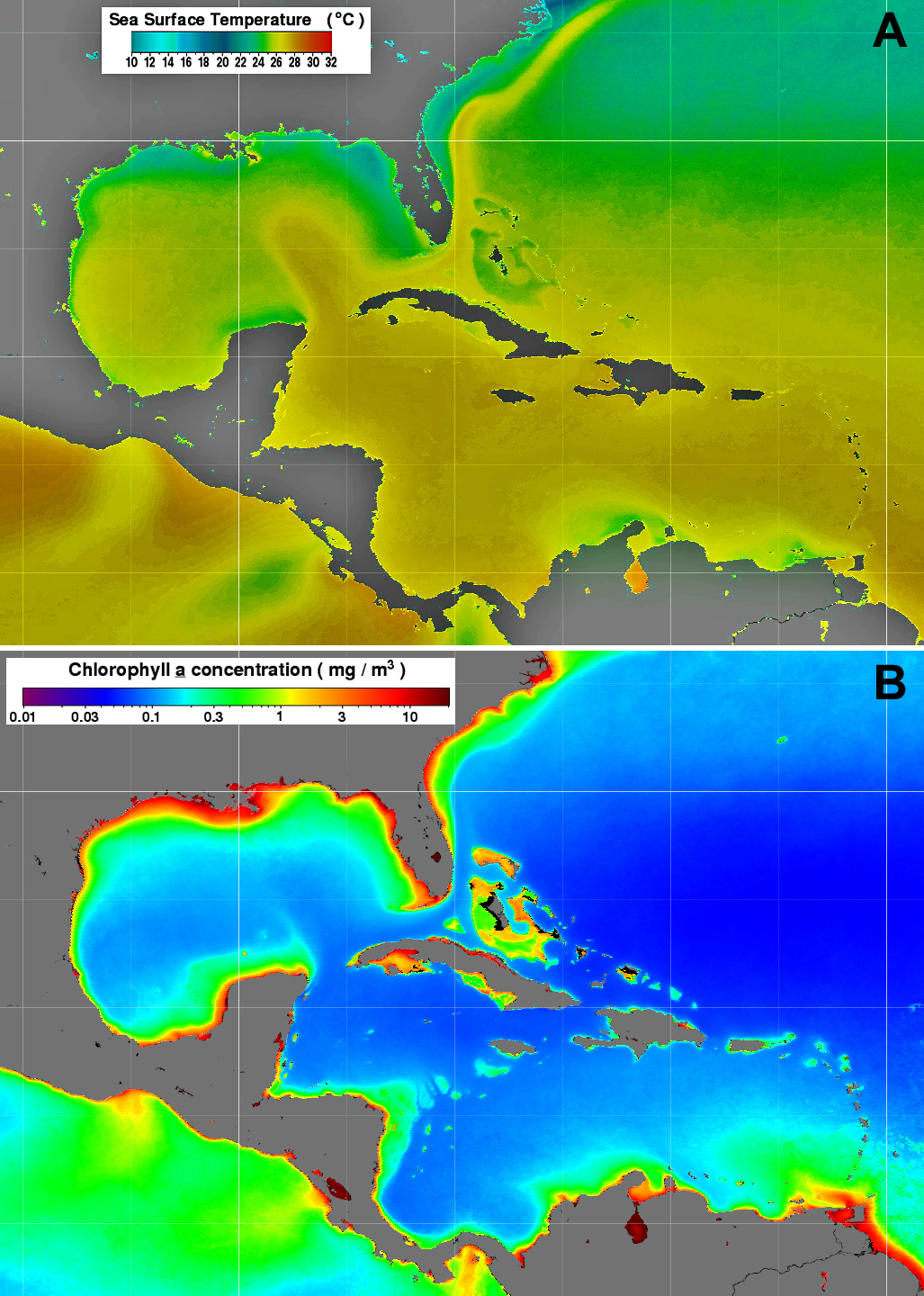

Supplement: Figure S8 — Average sea surface temperatures and chlorophyll concentrations in the study area. A: Average sea surface temperature (July 2002–October 2013), B: chlorophyll concentration (November 2011–October 2013). Source: Aqua MODIS data publically available at http://oceancolor.gsfc.nasa.gov/cgi/l3, accessed 2013 November 27. (TIF) [file pone.0102918.s008.tif]

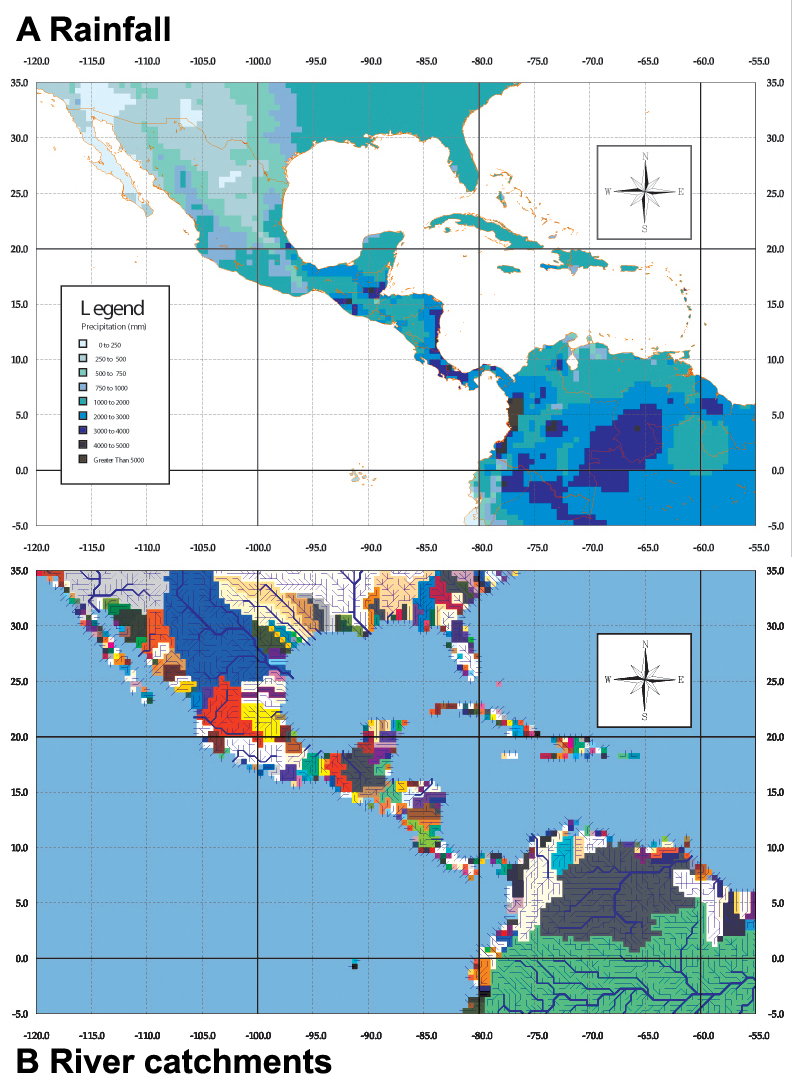

Supplement: Figure S9 — Rainfall patterns and river catchments in the study area. A: Distribution of rainfall, B: Distribution of river catchments throughout the study area. Images courtesy R. Lammers, Water Systems Analysis Group, University of New Hampshire. (TIF) [file pone.0102918.s009.tif]

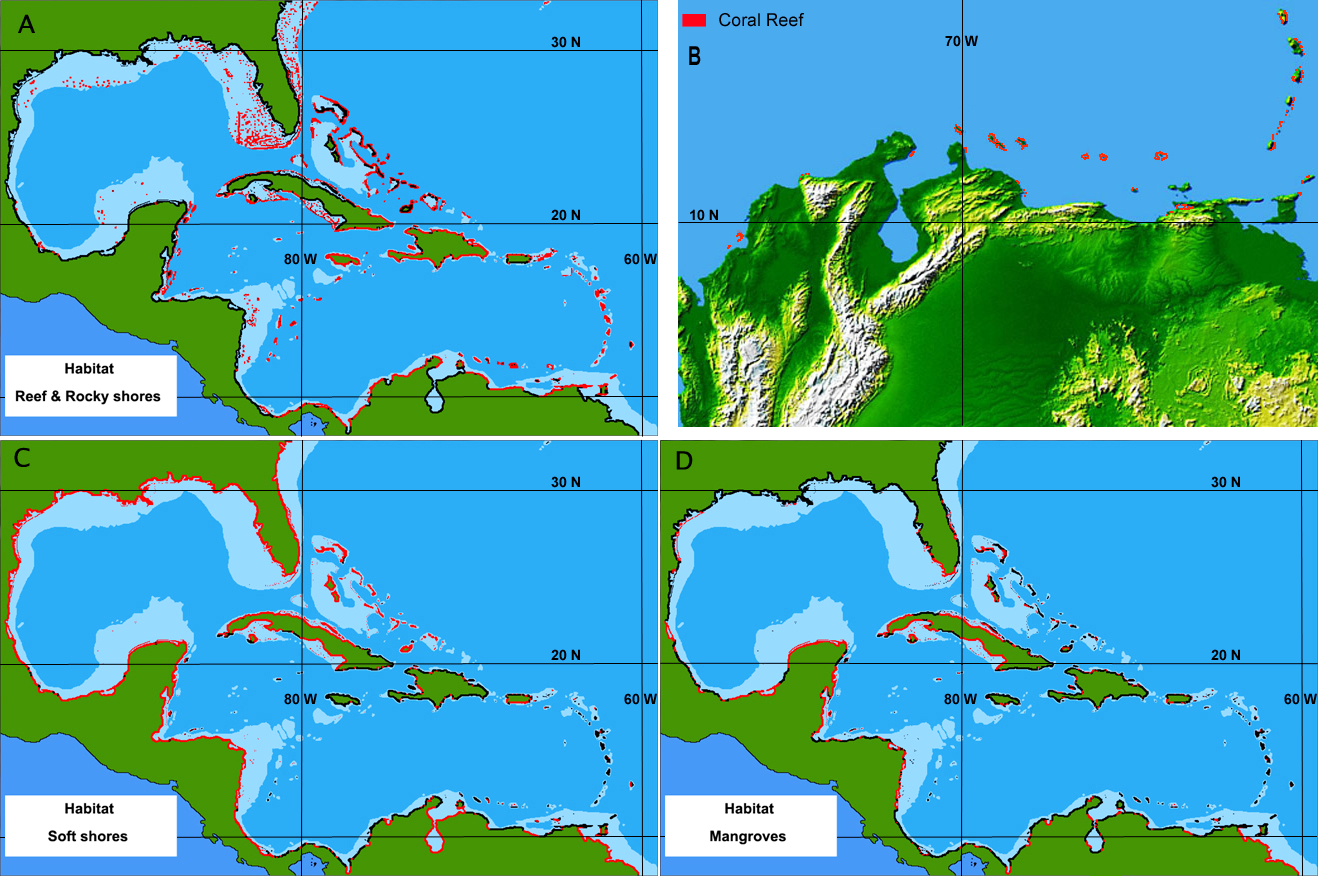

Supplement: Figure S10 — Habitat types in the study area. Schematic representations of distributions of different habitat types in the study area. Sources for A, C & D: (2–4); sero.nmfs.noaa.gov/hcd/pdfs/efhdocs/gom_efhhapc_poster. http://ocean.floridamarine.org; inspection of Google Earth images; B: Northern South America coral reef distribution after Maps 5f and 6e of ref (2). (TIF) [file pone.0102918.s010.tif]

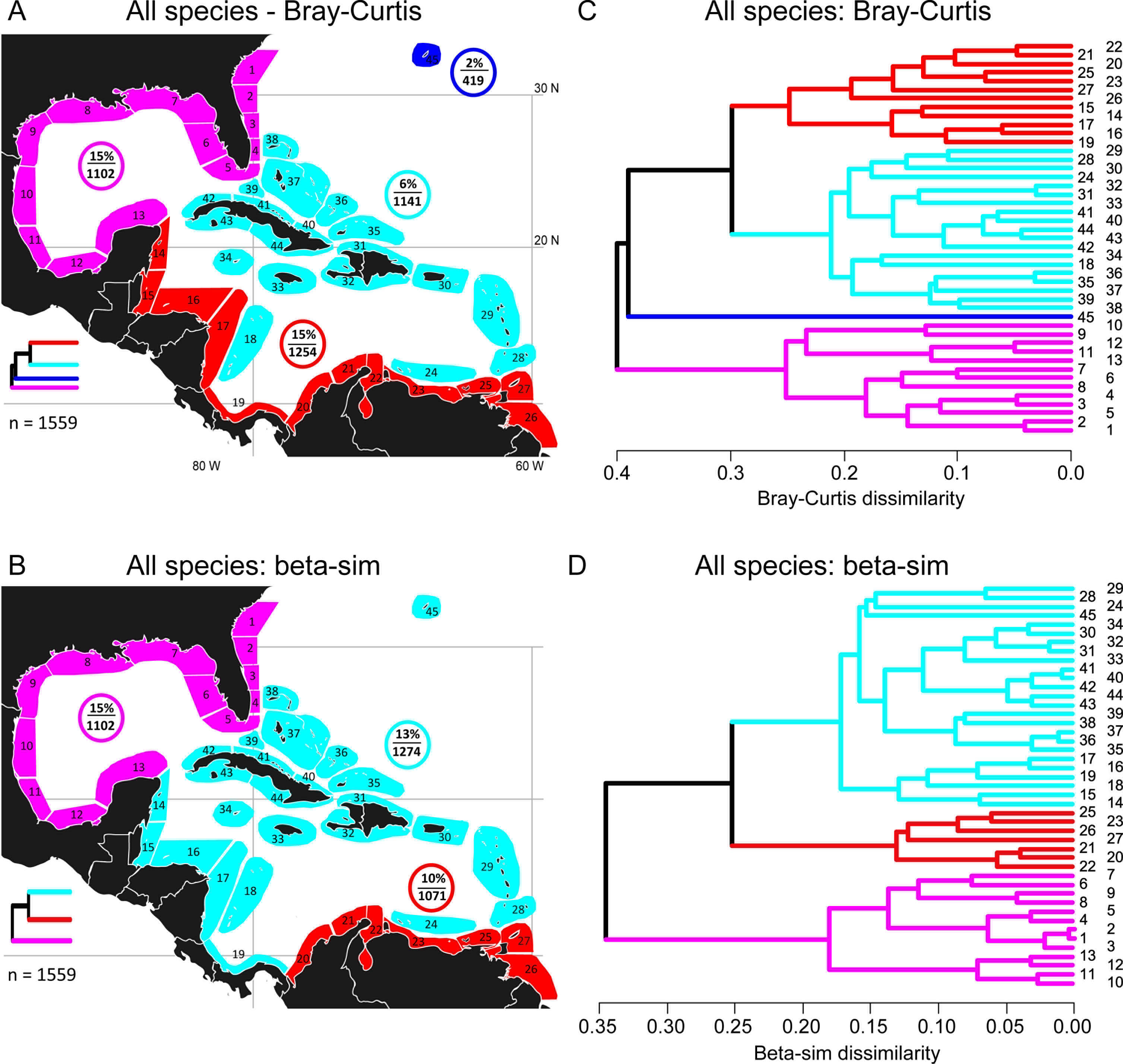

Supplement: Figure S11 — Major cluster configurations produced by different analytical methods. Optimal configuration of major clusters of sites in the study area based on dendrograms from Bray-Curtis/ANOSIM and beta-sim/evaluation curve analyses of the whole fauna (see methods). A: Bray-Curtis/ANOSIM cluster pattern, B: Beta-sim/evaluation cluster pattern, C: Bray-Curtis dendrogram, D: beta-sim dendrogram. %/n in colored circle indicates % of species unique to that cluster and no. species in the cluster; each dendrogram is a schematic based on the corresponding whole dendrogram, indicating relationships between the major clusters; n below schematic = total number of species. (TIF) [file pone.0102918.s011.tif]

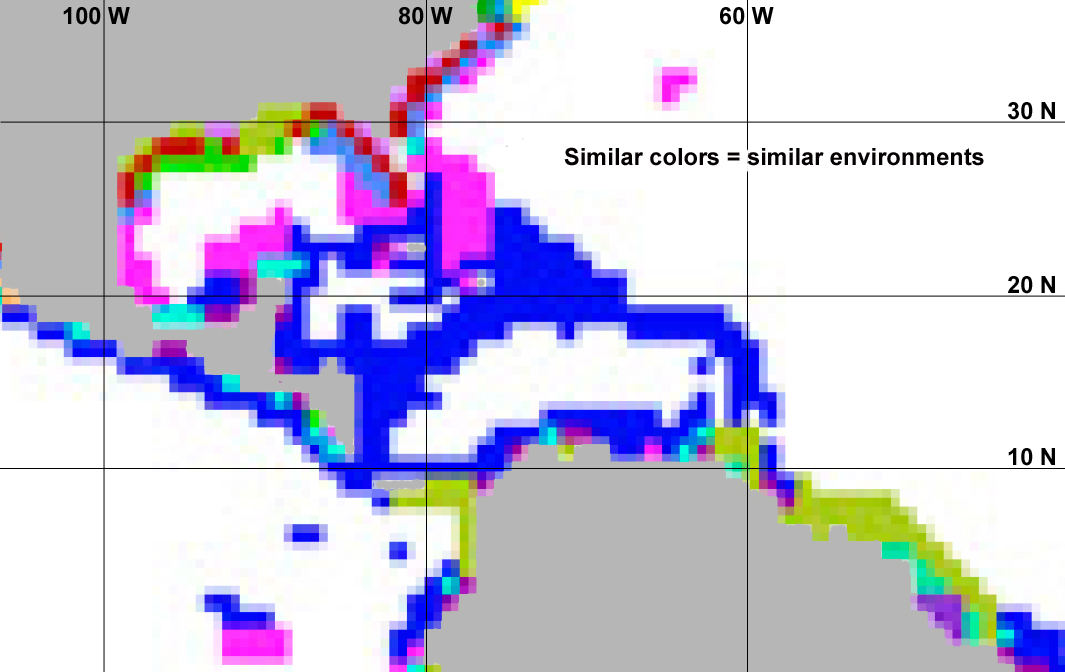

Supplement: Figure S12 — Environmental heterogeneity throughout the study area. Cells with similar regimes of primary productivity, sea surface temperature and salinity have similar colors, dissimilar cells have dissimilar colors. With permission, from Fig. 1E of (5). (TIF) [file pone.0102918.s012.tif]

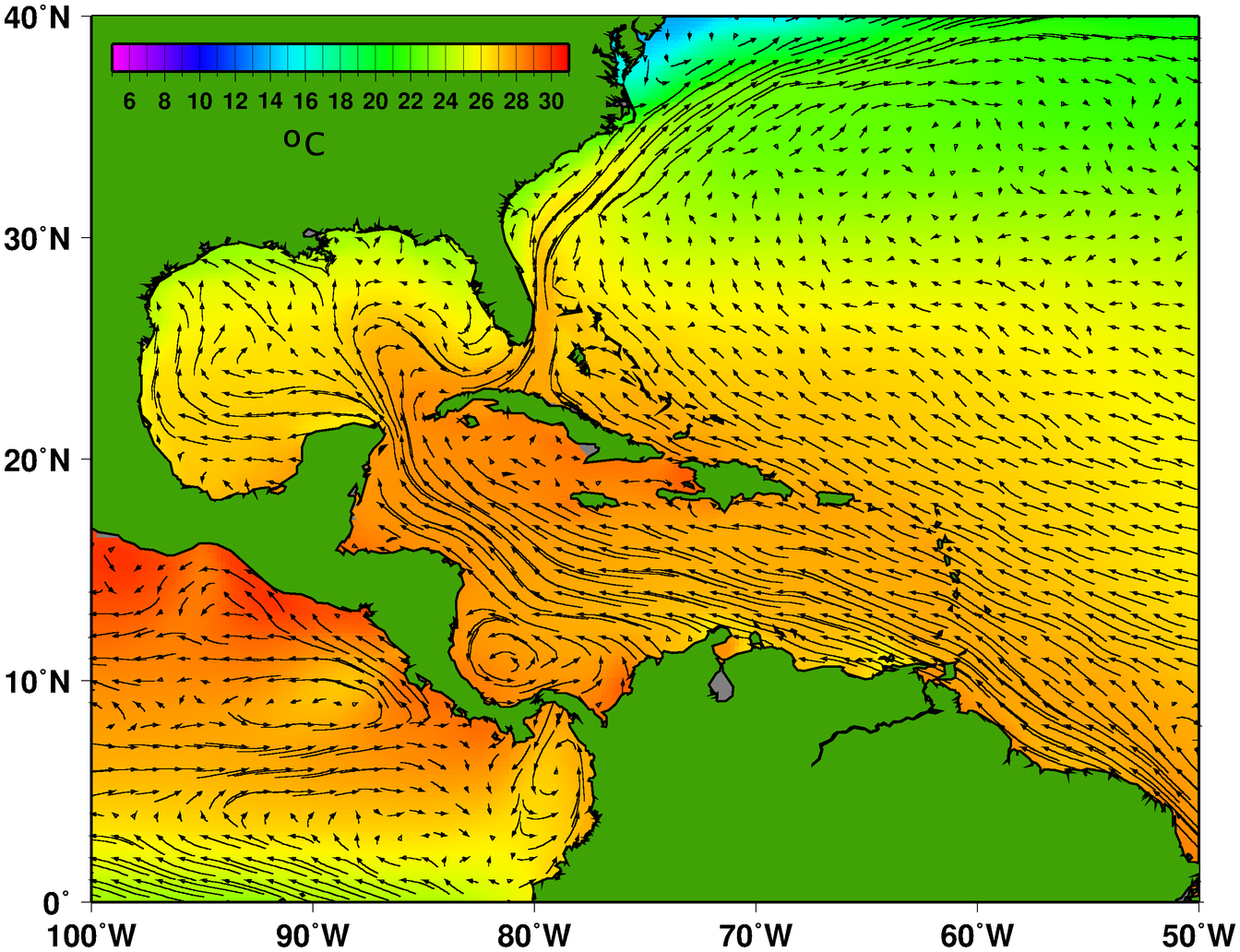

Supplement: Figure S13 — Surface ocean currents in the study area. Map courtesy of EH Ryan (eryan@rsmas.miami.edu). (TIF) [file pone.0102918.s013.tif]

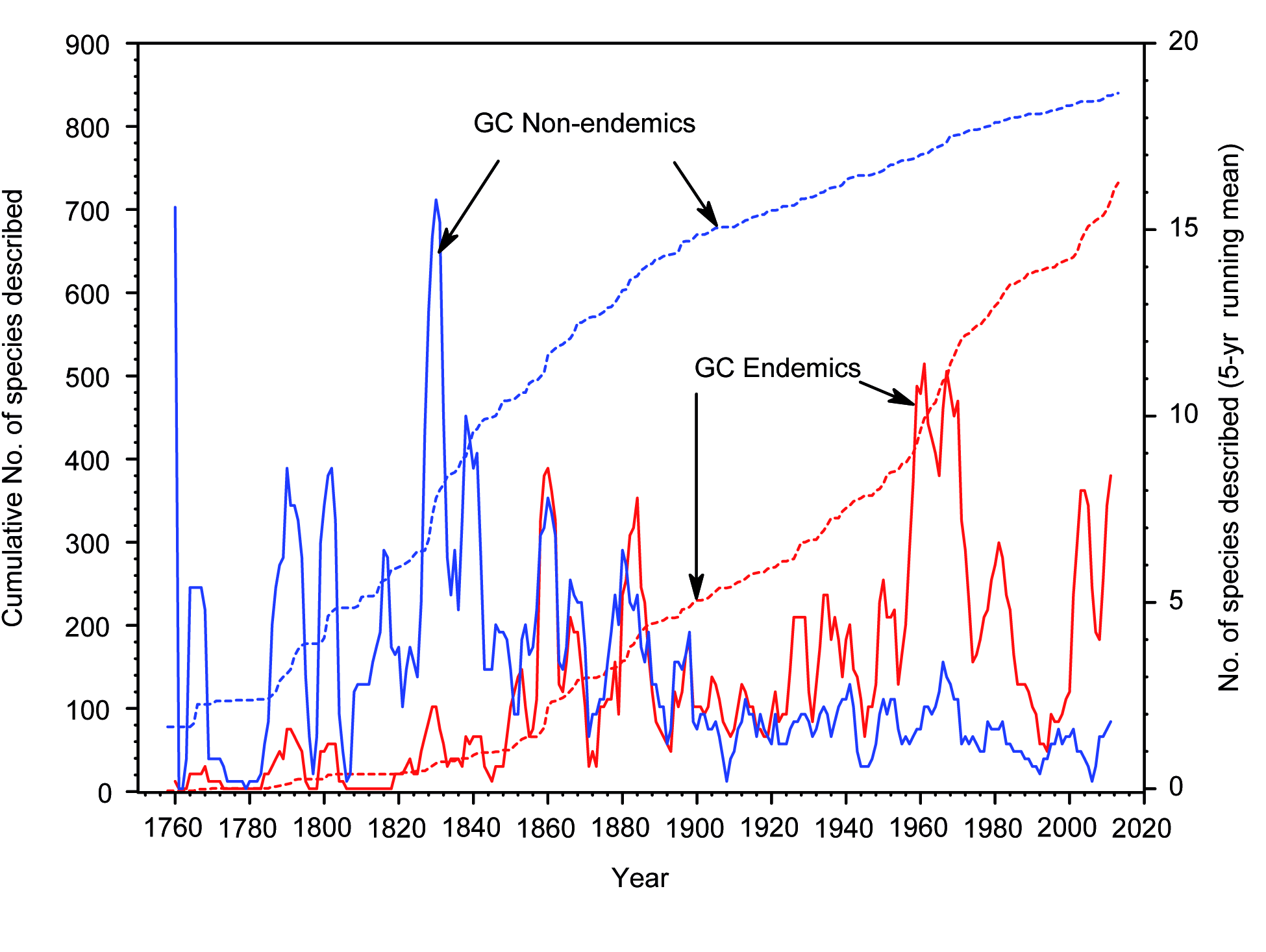

Supplement: Figure S14 — Accumulation of species descriptions of Greater Caribbean shorefishes. Accumulation curves and running means of rates of description per year for regional endemics and non-endemics. Source: F Zapata and DR Robertson, unpublished data. (TIF) [file pone.0102918.s014.tif]

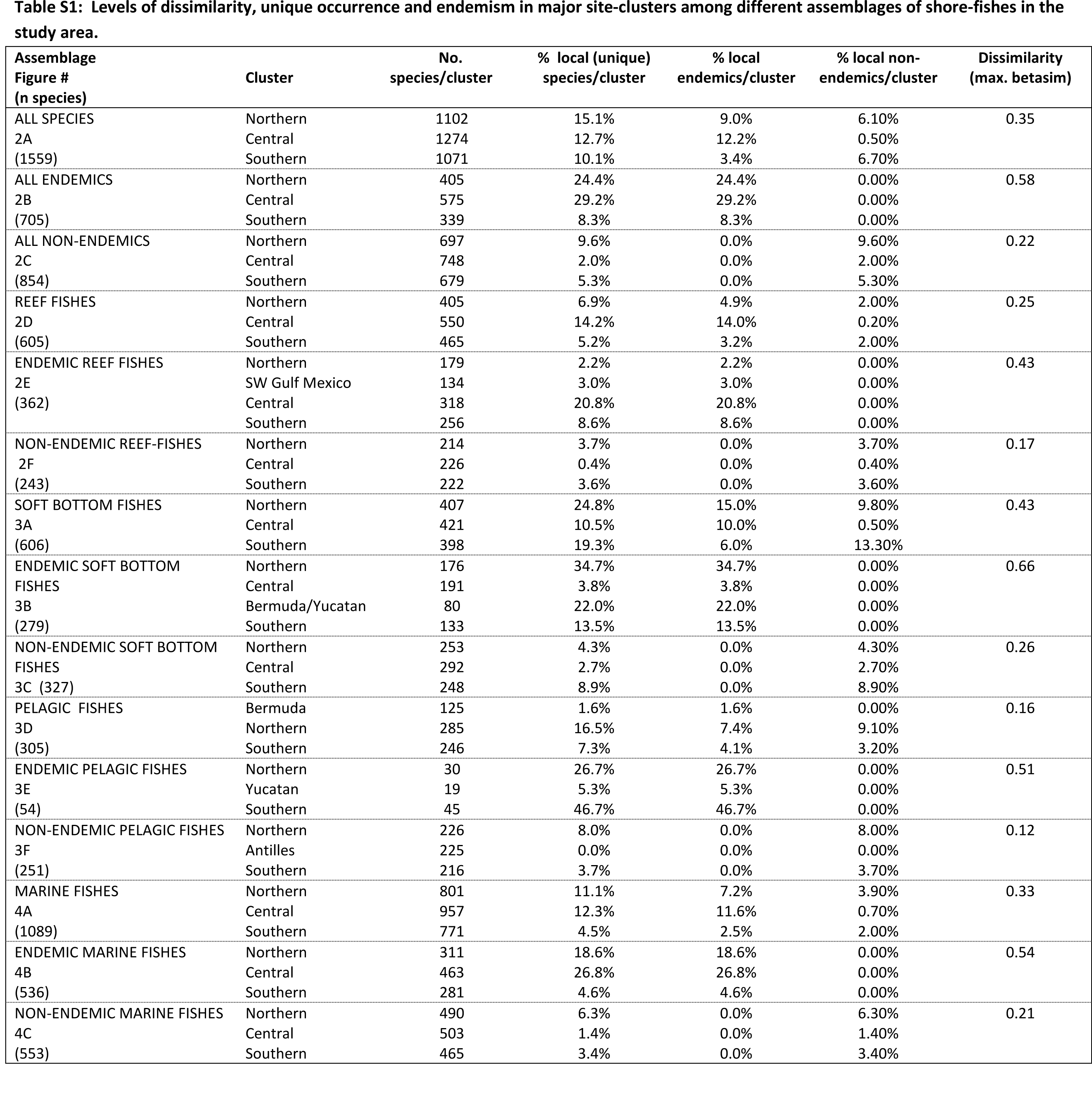

Supplement: Table S1 — Levels of dissimilarity, unique occurrence and endemism in major site-clusters among different assemblages of shorefishes in the study area. (TIF) [file pone.0102918.s015.tif]
