# Supplementary material for: Defining and Dividing the Greater Caribbean: Insights from the Biogeography of Shorefishes
Source: PLoS One. 2014 Jul 23;9(7):e102918. doi: 10.1371/journal.pone.0102918 (PMC4108436; doi:10.1371/journal.pone.0102918)
Supplement: Appendix S1 — Sources of data on shorefish distributions used to construct the species range-map database used in the analyses. (DOC) [file pone.0102918.s016.doc]

**Supporting Information**

**Appendix S1: Sources of data on shorefish distributions used to construct the species range-map database used in the analyses.**

1. **Online databases:**

These include not only databases from various aggregators (**FISHNET, FISHNET2, OBIS, GBIF** and **FISHBASE**), but also individual databases from the **Gulf Coast Research Laboratory;** the **US National Museum of Natural History;** the **American Museum of Natural History**; **Scripps Institution of Oceanography;** the **Natural History Museum, Los Angeles County**; the **Florida Museum of Natural History; INVEMAR (Colombia),**  the **Museo de Historial Natural Marina de Colombia, and** the **Sistema de Información sobre Biodiversidad de Colombia;** the **British Museum of Natural History;** the **Gulf of Mexico Fishnet; Marinespecies.org**;**,** the **US Geological Surve**y; **NOAA;** the **Swedish Museum of Natural History; the Muséum National d’Histoire Naturelle (France): the Danish Museum of Natural History; SEAMAP-Pascagoula;** la **Universidad de Costa Rica;** the **Florida Museum of Natural History,** and data on carefully selected (i.e. readily recognizable) species from expert visual surveys of fishes by the **Reef Environmental Education Foundation (REEF.org)** and **AGGRA** (the Atlantic and Gulf Rapid Reef Assessment) surveys of reef fishes (www.agrra.org).

1. **Information from individual researchers**:

C Baldwin (the Bahamas, Belize, Tobago); W Smith-Vaniz (Puerto Rico, the Virgin Is and Bermuda); B Victor (blennioids and gobiids), O Puebla (*Hypoplectrus* spp); A Polanco and A Acero (Colombia); R Betancur (Ariids); R Claro (Cuba). In addition DRR obtained site-record data from photographs of species (those that could be confidently identified) taken at known sites in the study area that he obtained through searches for images on the internet.

**3. General scientific literature providing information on fish distributions:**

**Bohlke JE, Chaplin CCG** (1968) Fishes of the Bahamas. Wynnewood: Livingston.

Carpenter KE (Ed). The living marine resources of the western central Atlantic. Vol 1: Introduction, molluscs, crustaceans, hagfishes, sharks, batoid fishes, and chimaeras. Rome: FAO.

Carpenter KE (Ed). The living marine resources of the western central Atlantic. Vol 2: Bony fishes part 1 (Acipenseridae to Grammatidae). Rome: FAO.

Carpenter KE (Ed). The living marine resources of the western central Atlantic. Vol 3: Bony fishes part 2 (Opistognathidae to Molidae) sea turtles and marine mammals. Rome: FAO.

Cervigon F (1991) Los Peces Marinos de Venezuela. Vol 1. Caracas: Fundación Científica Los Roques.

Cervigon F (1993) Los Peces Marinos de Venezuela*.* Vol 2. Caracas: Fundación Científica los Roques.

Cervigon F (1994) Los Peces Marinos de Venezuela*.* Vol 3 Caracas: Editorial Ex Libris.

Cervigon F (1996) Los Peces Marinos de Venezuela. Vol 4. Caracas: Editorial Ex Libris.

Cervigon F, Alcalá A (1999) Los Peces Marinos de Venezuela*.* Vol 5. Estado Nueva Esparta: Fundación Museo del Mar.

Claro R, Lindeman KC, Parenti LR 2001. Ecology of the marine fishes of Cuba. Washington DC: SI Press.

**Colin PL** 1975. Neon gobies. Neptune City: TFH.

Fishes of the Western North Atlantic. 1948 Memoirs of the Sears Foundation of Marine Research Mem. 1 (pt 1). New Haven: Sears Foundation.

Fishes of the Western North Atlantic. 1953 Memoirs of the Sears Foundation of Marine Research Mem. 1 (pt 2). New Haven: Sears Foundation.

Fishes of the Western North Atlantic. 1963Memoirs of the Sears Foundation of Marine Research. Mem. 1 (pt 3). New Haven: Sears Foundation.

Fishes of the Western North Atlantic. 1964 Memoirs of the Sears Foundation of Marine Research. Mem. 1 (pt 4). New Haven: Sears Foundation.

Fishes of the Western North Atlantic. 1966 Memoirs of the Sears Foundation of Marine Research. Mem. 1 (pt 5). New Haven: Sears Foundation.

Fishes of the Western North Atlantic. 1973 Memoirs of the Sears Foundation of Marine Research. Mem. 1 (pt 6). New Haven: Sears Foundation.

Fishes of the Western North Atlantic. 1982 Memoirs of the Sears Foundation of Marine Research. Mem. 1 (pt 8). New Haven: Sears Foundation.

Fishes of the Western North Atlantic. 1989 Memoirs of the Sears Foundation of Marine Research. Mem. 1 (pt 9). New Haven: Sears Foundation.

**Kells V, Carpenter K** (2011). A field guide to coastal fishes from Maine to Texas. Baltimore: Johns Hopkins U Press.

McEachran JD, Fechhelm JD (1998) Fishes of the Gulf of Mexico. Vol 1: Myxiniformes to Gasterosteiformes. Austin: U Texas Press.

McEachran JD and Fechhelm JD (2005) Fishes of the Gulf of Mexico. Vol 2: Scorpaeniformes to Tetraodontiformes. Austin: U Texas Press.

Munroe TA (1998). Systematics and ecology of tonguefishes of the genus *Symphurus* (Cynoglossidae, Pleuronectiformes) from the western Atlantic ocean. Fish Bull 96: 1-182.

**Pietsch TW, Grobecker** DB (1987) Frogfishes of the world: systematics, zoogeography, and behavioral ecology. Stanford: Stanford U Press,

**Randall** JE (1998) Caribbean reef fishes. 3^rd^ Ed. Neptune City: TFH.

Smith CL, Tyler JC, Davis WP, Jones RS, Smith DG, Baldwin CC (2003) The fishes of pelican cays, Belize. Atoll Res Bull 497: 1-88.

Smith-Vaniz WF, Collette BB, Luckhurst BE (1999). Fishes of Bermuda: history, zoogeography, annotated checklist, and identification keys. Lawrence: Allen Press.

1. **Data availability**

A list of species included in this analysis can be found in: Robertson DR, Van Tassell J (2012). Fishes: Greater Caribbean. An Identification Guide to the shore-fish fauna of the Caribbean and adjacent areas. IOS App version 1.0 (October 23, 2012) for iPhone. <https://itunes.apple.com/us/app/fishes-greater-caribbean/id570048678?mt=8> . Maps of species ranges in that iphone App are approximate only, and were not used in the present analysis. More detailed maps that include georeferenced site records and that were used in the present analysis will be included in the future iPad version of that app and a website version. These procedures are equivalent to those by which we made similar data available following publication of our equivalent analysis of the biogeography Tropical Eastern Pacific fishes [19], in both a website (Robertson DR, Allen GR (2008) Shorefishes of the tropical Eastern Pacific online information system. www.stri.org/sftep ), and an iOS app for the iPad (Robertson DR, Allen GR (2012) Fishes: East Pacific. An Identification Guide to the shore-fish fauna of the tropical eastern Pacific. IOS App version 1. 0 (January 19, 2012) for iPhone and version 2.1 (0ctober 18, 2012) for iPad/iPhone. <https://itunes.apple.com/us/app/fishes-east-pacific/id494644648?mt=8>)

**Table S1: Levels of dissimilarity, unique occurrence and endemism in major site-clusters among different assemblages of shorefishes in the study area.**

**Supporting Figures S1-S14**

**References for supporting figures.**

1. Smith ML, Carpenter KE, Waller RW (2002) An introduction to the oceanography, geology, biogeography, and fisheries of the tropical and subtropical western central Atlantic. In: Carpenter KE, editor. The living marine resources of the western central Atlantic. Vol 1. Rome: FAO. 1-23.

2. Spalding MD, Ravilious C, Green EP (2001) World Atlas of Coral Reefs, Berkeley: U California Press.

3. Galtsoff PS (1954). Gulf of Mexico, its origin, waters and marine life. US Fish Bull 55: 1-605.

4. Rezak R, Bright TJ, McGrail DW (1985) Reefs and banks of the northwestern Gulf of Mexico, the geological, biological and physical dynamics. New York: J Wiley.

5. Belanger CL, Jablonski D, Roy K, Berke SK, Krug AZ, Valentine JW (2012). Global environmental predictors of benthic marine biogeographic structure. PNAS 109: 14046-14051.

**Supporting Figure Captions**

**Figure S1.** **Distribution of species occurrence records in the study area.**

Combined plot of georeferenced site records for all species used in the construction of the detailed species range maps included in the analyses. Sources of records in Appendix S1. Note: this figure also includes (a small number of) records for non-resident species, which were not used in the analyses.

**Figure S2.** **Example evaluation curve used to determine major cluster configurations.**

Evaluation curve demonstrating L method for finding the inflexion point of the curve (see methods) to establish optimal number of major clusters for the whole fauna assemblage. Local species = species found only in a particular cluster.

**Figure S3.** **Hierarchical cluster dendrogram of beta-sim dissimilarities between the 45 site faunas: all species and all reef fishes.**

A-C: all species, all endemic species, all non-endemic species; D-F: all reef fishes, endemic reef fishes, non-endemic reef fishes.

**Figure S4**. **Hierarchical cluster dendrogram of beta-sim dissimilarities between the 45 site faunas: soft bottom and pelagic fishes.**

A-C: all soft bottom species, endemic soft bottom species, non-endemic soft bottom species; D-F: all pelagic fishes, endemic pelagic fishes, non-endemic pelagic fishes.

**Figure S5.** **Hierarchical cluster dendrogram of beta-sim dissimilarities between the 45 site faunas: marine and non-marine fishes.**

A-C: all marine species, endemic marine species, non-endemic marine species; D-F: all non-marine species, endemic non-marine species, non-endemic non-marine species.

**Figure S6.** **Distribution of shelf fishes also found in areas to the north and south of the Greater Caribbean.**

A: Species found further north, B: species found further south; Figs 14 and 15 of ref [1].

**Figure S7.** **Major cluster configurations for shallow and deep species.**

Optimal configuration of major clusters of sites based on beta-sim dissimilarity dendrograms and defined by evaluation curve fitting (see methods). A: Species restricted to 20m depth or shallower; B species whose depth ranges extend below 50m. C & D endemic subsets of A & B respectively. %/n in colored circle indicates % of species unique to that cluster and no. species in the cluster; each dendrogram is a schematic based on the corresponding whole dendrogram (not shown) that indicates relationships between the major clusters; n below schematic = total number of species.

**Figure S8. Average sea surface temperatures and chlorophyll concentrations in the study area.**

A: Average sea surface temperature (July 2002-October 2013), B: chlorophyll concentration (November 2011- October 2013). Source: Aqua MODIS data publically available at <http://oceancolor.gsfc.nasa.gov/cgi/l3>, accessed November 27, 2013.

**Figure S9.** **Rainfall patterns and river catchments in the study area.**

A: Distribution of rainfall, B: Distribution of river catchments throughout the study area. Images courtesy R. Lammers, Water Systems Analysis Group, University of New Hampshire.

**Figure S10.** **Habitat types in the study area.**

Schematic representations of distributions of different habitat types in the study area. Sources for A, C & D: (2-4); sero.nmfs.noaa.gov/hcd/pdfs/efhdocs/gom_efhhapc_poster. [http://ocean.floridamarine.org](http://ocean.floridamarine.org/); inspection of Google Earth images; B: Northern South America coral reef distribution after Maps 5f and 6e of ref [2].

**Figure S11. Major cluster configurations produced by different analytical methods**

Optimal configuration of major clusters of sites in the study area based on dendrograms from Bray-Curtis/ANOSIM and beta-sim/evaluation curve analyses of the whole fauna (see methods). A: Bray-Curtis/ANOSIM cluster pattern, B: Beta-sim/evaluation cluster pattern, C: Bray-Curtis dendrogram, D: beta-sim dendrogram. %/n in colored circle indicates % of species unique to that cluster and no. species in the cluster; each dendrogram is a schematic based on the corresponding whole dendrogram, indicating relationships between the major clusters; n below schematic = total number of species.

**Figure S12.** **Environmental heterogeneity throughout the study area.**

Cells with similar regimes of primary productivity, sea surface temperature and salinity have similar colors, dissimilar cells have dissimilar colors. With permission, from Fig 1E of [5].

**Figure S13.** **Surface ocean currents in the study area.**

Map courtesy of EH Ryan ([eryan@rsmas.miami.edu](mailto:eryan@rsmas.miami.edu))

**Figure S14. Accumulation of species descriptions of Greater Caribbean shorefishes.**

Accumulation curves and running means of rates of description per year for regional endemics and non-endemics. Source: F Zapata and DR Robertson, unpublished data.
